# Functional Passenger-Strand miRNAs in Exosomes Derived from Human Colon Cancer Cells and Their Heterogeneous Paracrine Effects

**DOI:** 10.7150/ijbs.40787

**Published:** 2020-02-04

**Authors:** Qingkun Gao, Fuming Lei, Qingmin Zeng, Zhaoya Gao, Pengfei Niu, Junnan, Ning, Jie Li, Jun Zhang

**Affiliations:** 1 Department of General Surgery, Beijing Friendship Hospital, Capital Medical University, Beijing 100050. China; 2 Department of General Surgery, Gastrointestinal Surgery, Peking University Shougang Hospital, Beijing 100144, China; 3 Research Department, Genex Health Co., Ltd, Beijing 100195, China

**Keywords:** colon cancer, exosome, passenger strand, miRNA*, integrin

## Abstract

Exosome-mediated microRNAs (miRNAs) are closely related to the occurrence, development, invasion, metastasis, therapeutic resistance, diagnosis and treatment of malignant tumors. Guide-strand miRNA and passenger-strand miRNA (miRNA*) exist in miRNA processing, but the function of passenger-strand miRNA is often overlooked. In this study, we attempted to identify functional miRNA*s in exosomes derived from human colon cancer SW620 cells. miRNA expression profiles of human normal colonic epithelial cells NCM460 and colon cancer cells SW620 were compared by high-throughput sequencing. According to the sequencing results, we defined two sets of differentially expressed miRNAs: “high in exosome and high in cell” (HEHC) and “high in exosome but low in cell” (HELC). As passenger-strand miRNAs, miR-2277-3p and miR-26b-3p, which belong to different sets, have diametrically opposite functions. MiR-2277-3p promotes proliferation, migration, and invasion of SW620 cells by targeting NUPR1L, while miR-26b-3p exerts an inhibitory effect by targeting PFDN1. Using exosomes as transport vectors, the effect of exosomes rich in miR-2277-3p on cells is consistent with the effect of liposome-transfected overexpressed miR-2277-3p, resulting in a cancer-promoting effect. However, exosomes rich in miR-26b-3p did not have a tumor suppressor effect. Further analysis revealed that exosomes rich in miR-2277-3p also had a high abundance of integrin β4. Altering the abundance of integrin β4 in exosomes changes the ability of exosomes to be taken up by cells, thereby altering the paracrine effects of exosomes. In summary, we revealed the fact that a large number of passenger-strand miRNAs exist in exosomes of colon cancer cells, these miRNAs are preliminarily categorized into two sets, and miR-2277-3p and miR-26b-3p, as representatives of each set, showed opposite functions. In addition, we revealed that integrin β4 is a marker of exosome heterogeneity in colon cancer cells, which directly correlates with the ability of exosomes to be uptaken by cells of the same kind, thus regulating the paracrine effect of exosomes.

## Introduction

Exosomes are small lipid bilayer-enclosed vesicles secreted by cells to the extracellular space. They are 30-100 nm in diameter and contain complex biological molecules, including amino acids, proteins, nucleic acids (mRNAs, microRNAs, DNAs, etc.), and many cellular metabolites 1, 2. Many studies have shown that these biomolecules within exosomes are biologically active. Exosomes exist not only in the vicinity of tissue that secreted them but also can be transported far away in blood, thus participating in both local and systematic communication 3-6. Unlike many cytokines that are directly dissolved in blood, biomolecules in exosomes are well protected and concentrated 7, 8. In recent years, cancer exosomes have been explored for their important role in tumor progression, especially inflammatory response, formation of pre-metastatic niche, and organotropism that play key roles in cancer metastasis 9-12.

MiRNAs are highly conserved small RNA molecules, 19-23 nucleotides (nt) in length, that do not encode proteins but usually regulate the expression of mRNAs at the post-transcriptional level. Some miRNAs circulate stably in blood without being degraded by RNases, because they are either complexed with circulating proteins or encapsulated by circulating exosomes. Studies have shown that circulating miRNAs inside exosomes are highly stable - their patterns showed no significant changes after being stored at in-depth ℃ for 96 h or at -70 ℃ for 1 month. Among all RNA in exosomes, mature miRNAs accounted for 41.7% 13. Gallo et al. have proved that exosomes are rich in miRNAs and can be used as a reliable raw material for miRNA research 14. Exosomes not only protect miRNAs from degradation *in vitro* but also transport miRNAs to specific cells to exert their regulatory effects. Exosomes secreted by tumor cells, which often contain a large amount of miRNAs, can play a major role in the self-regulation of tumor cells and have now become a focus of cancer research 15-17.

During the processing of miRNAs, the precursor miRNA (pre-miRNA) is usually cleaved by the Dicer enzyme to form a small double-stranded RNA of about 22 nt in length. The strand complementary to the target mRNA is called the guide strand (miRNA), and the other strand is called the passenger strand (miRNA*). Previous studies have largely focused on the guide strand, while functions of the passenger strand were largely overlooked. Recently, more and more studies have suggested that the passenger strand also plays an important role in the regulation of gene expression. The passenger strand not only promotes the assembly of the RNA induced silence complex (RISC) but also is incorporated into the RISC, exerting gene-silencing effects itself or assisting the leader strand or other miRNAs in the regulation of corresponding genes 18-20. Bang et al. reported, for the first time, that exosomes of cardiac fibroblasts contain a large number of passenger-strand miRNAs and proved that miR-21* (miR-21-3p), as a paracrine RNA molecule, can effectively induce cardiomyocyte hypertrophy 21. This provides a new perspective for the functional study of passenger-strand miRNAs.

However, to date, there have been no systematic studies on functional passenger-strand miRNAs in tumor exosomes. In this study, we used human colon cancer cells as a model to preliminarily investigate the distribution of passenger-strand miRNAs in colon cancer exosomes and the paracrine effects of functional passenger-strand miRNAs.

## Materials and methods

### Clinical specimens

Healthy individuals (n=20) and consenting patients with CRC (n=20) were enrolled at the Department of General Surgery of Peking University Shougang Hospital upon approval from the research ethics committee. Blood samples were collected at diagnosis (before the operation; baseline). Clinicopathological features are listed in Supplementary Table S1. Peripheral blood (15 ml) was collected in tubes containing disodium EDTA (BD Diagnostics, Franklin Lake, NY, USA) and processed to obtain plasma through centrifugation at 2,000 g for 15 min at 4 ℃ not later than 4 h after withdrawal.

### Cell culture and transfection

The human colorectal cancer cell line SW620 and human normal colonic epithelial cell line NCM460 were obtained from the Cell Resource Center, Peking Union Medical College. Cells were cultured in DMEM (Invitrogen, Carlsbad, CA, USA) containing 10% (v/v) fetal bovine serum (FBS; Invitrogen), 100 mg/ml streptomycin sulfate, and 100 U/ml penicillin G (Sigma-Aldrich) at 37 ℃ with 5% CO_2_. Cells were seeded in 6-well or 24-well plates, respectively, and transfected with miRNA (10-100nM), siRNA (10nM), or vectors using Lipofectamine2000 (Invitrogen) according to the manufacturer's recommendations. The plasmid pCMV-NUPR1L and pCMV- PFDN1 were obtained from OriGene (OriGene Technologies, USA). Integrin β4 (ITGB4) siRNA (s7584) and Negative Control siRNA (AM4611) were obtained from Thermo Fisher Scientific. The miRNA mimic sequences were synthesized by GenePharma.

### Luciferase assay

The human *NUPR1L* 3′ UTR (GenBank accession NM_001145712.2) and *PFDN1* 3′ UTR (GenBank accession NM_002622.5) containing the predicted miRNA binding sites were PCR amplified using genomic DNA from SW620 cells. The PCR product was then cloned into the pGL3 vector (Promega, Madison, WI, USA) located between *Xba*I and *EcoR*I restriction enzyme sites downstream of the firefly luciferase reporter gene. MutanBEST Kit (Takara, Japan) was used to mutate the target binding sites of miRNA and cloned into the pGL3 vector.

The firefly luciferase reporter plasmids containing wt or mutant 3'UTRs (100ng), the control renilla luciferase plasmid (pRL-CMV, 5ng) (Promega), and miRNA mimics were co-transfected into the cells using Lipofectamine2000 according to the manufacturer's protocol. After 48 hours, luciferase activities were measured using the Dual-Luciferase Reporter Assay System (Promega).

### Exosomes extraction

SW620 cells were cultured in RMPI-1640 supplemented with 10% exosome-depleted FBS for 48h. Exosomes in the culture supernatant were isolated using the ExoQuick-TC kit (System Biosciences, Mountain View, CA, USA) as described by the manufacturer. In general, the culture medium was collected and centrifuged at 3,000 g for 15 min. The supernatant was filtered through a 0.22μm PVDF filter (Millipore). The appropriate volume of the Exoquick Exosome Precipitation Solution was added to the filtered culture medium and mixed well by inverting. After 12 h of refrigeration, the mixed liquid was centrifuged at 10,000 g for 30 minutes, and the supernatant was discarded. The separated Exosome granules were re-suspended in PBS, stored at -80 ℃ or used directly. Relative quantification of exosomes was performed using the EXOCET Exosome Quantitation Kit (System Biosciences). The methods of RNA and protein extraction from exosomes refer to those described in the above cell experiments.

To prepare miRNA*-rich exosomes, 100 μM miRNA* was transfected into SW620 cells, and 12 hours later, culture supernatants were collected to extract exosomes for use.

Plasma samples from different donors were thawed in a room temperature water bath until completely liquid, then centrifuged at 2,000 g for 30 minutes to remove any cell debris. The supernatant containing the cell-free serum was transferred to a fresh container. Each serum sample was then mixed with a 1/5 volume of total exosome separation reagent (Invitrogen), and then mixed well by vortexing until a homogenous solution was formed. Incubated at 4 ℃ for 30 min, centrifuged at 10,000g at room temperature for 10 min, the supernatant was absorbed and discarded, exosome particles were re-suspended in PBS buffer, and stored at -80 ℃ or used directly.

### Transmission electron microscopy (TEM)

Exosomes were diluted to 0.5 mg/ml suspension with 1×PBS at 4 ℃. Put the carbon-coated nickel mesh on the filter paper, absorbed the 20 µl exosomes suspension, and dropped onto the mesh. After 5min of baking under an infrared lamp, added 1-2 drops of 2% phosphotungstic acid (PTA) and baked for 5 min again. The morphology of exosomes was observed under the transmission electron microscope (JEM-1230, Jeol Ltd, Japan).

### RNA sequencing

Total RNA was isolated from exosomes using SeraMir Exosome RNA Purification Column Kit (SBI, RA808A-1) according to the manufacturer's protocol. The concentration of total RNA was measured using a Qubit®RNA Assay Kit with a Qubit®2.0 Fluormeter (Life Technologies) and the quality of total RNA was verified using an Agilent 2100 Bioanylzer RNA 6000 Pico Kit (Agilent Technologies).

For small RNA libraries, a total amount of 20 ng RNA per sample was used as input material to generate a small RNA library for the miRNAs analysis. Sequencing libraries were generated using NEBNext® Multiplex Small RNA Library Prep Set for Illumina (NEB) following the manufacturer's recommendations. Briefly, 3′ and 5′ adaptors were ligated along with RNA sample. The adaptor-ligated RNA fragments were reverse transcribed and PCR amplified with the unique index sequence. The libraries obtained from different samples were performed on a HiSeq 2500 (Illumina Inc.) and 50-bp reads were generated.

In order to analyze the expression and distribution of small RNA tags on reference sequences, we used Bowtie 22 to map small RNA tags to the reference sequences without any mismatch. Taking MiRBase20.0 as a reference, the potential miRNA and secondary structure were obtained by using improved mirdeep2 23 and srna-tools-cli software. Used a custom script to get the miRNA counts and the base deviation of the first position of the identified miRNA of a specific length and each location of all recognized miRNAs.

### Quantitative RT-PCR (QRT-PCR)

Total RNA was extracted with TRIzol reagent (Invitrogen). RNA samples were quantified at 260nm by spectrophotometry (GeneQuant, GE Healthcare, Piscataway, USA) and cDNA was produced by reverse transcription using miScript RT II Buffer (QIAGEN Inc., UK). The mixture was incubated at 37˚C for 60 minutes, then at 95 ˚C for 5 minutes. Real-time PCR was performed using miScript HiSpec Buffer (QIAGEN) and ABI 7500 Real-Time PCR System (Applied Biosystems, Warrington, UK). Gene expression assays were performed in triplicate and data were processed using the 2^-ΔΔ^CT method and normalized control. GAPDH was used as endogenous control.

MiRNAs were quantified using an All-in-One miRNA qRT-PCR test kit (GeneCopoeia, Rockville, MD, USA). For single-step cDNA synthesis, we used poly (A) polymerase to add the poly (A) tail to the 3′ends of the miRNAs, and reverse transcribed the poly (A) miRNAs using M-MLV reverse transcriptase and a unique oligo (dT) adapter primer. Then using universal joint PCR primers and miRNA-specific primers, an All-in-One qPCR Mix was used to detect specific reverse transcription miRNAs. Intracellular miRNAs were normalized against U6 snRNA. Cel-mir-39 was used as the exogenous control for miRNAs in exosomes. Primers for RT-qPCR are as follows (from 5′ to 3′): NUPR1L: 5'- CTACTACCTGCGCGACTTCC-3' (forward), 5'-ACTTCGAACAGGTCCTTCGG-3' (reverse); PFDN1: 5'-TGGAGCGAAGCGTTAAGGAA-3' (forward), 5'-CGGGTAAAAACTCCAGGGCT-3' (reverse); GAPDH: 5'-ACAACTTTGGTATCGTGGAAGG-3' (forward), 5'-GCCATCACGCCACAGTTTC-3' (reverse); Universal Adaptor primer, 5'-GCGAGCACAGAATTAATACGAC-3'; miR-2277-3p: 5'-CAGCGCCCTGCCT-3' (forward), 5'-CCAGTTTTTTTTTTTTTTTGAGCCA-3' (reverse); miR-26b-3p: 5'-CAGCCTGTTCTCCATTACTTG-3' (forward), 5'-GGTCCAGTTTTTTTTTTTTTTTAGC-3' (reverse); U6: 5'-CTCGCTTCGGCAGCACA-3' (forward), 5'- GCGAGCACAGAATTAATACGAC-3' (reverse); Cel-miR-39: 5'-GTCACCGGGTGTAAATCAG-3' (forward), 5'-GGTCCAGTTTTTTTTTTTTTTTCAAG-3' (reverse); Primers for other miRNA* qRT-PCR are shown in Table S2.

### Protein extraction and western blot analysis

ProteoJET™ Mammalian Cell Lysis Reagent (Thermo Scientific, Waltham, MA) was added to extract the cell protein of each group, and the protein was quantified by the BCA method. Proteins (50 μg) were separated using 10% SDS-PAGE and transferred to PVDF membranes. The PVDF membranes were blocked with 5% non-fat dry milk in TBST for 1 hour, then probed with the specific primary antibodies at 4 ℃ for 24 h, and incubated with the second antibody at room temperature for 1 h. Finally, ECL developer (Thermo Scientific) was added and exposure imaging was performed by Odyssey gel imaging system. Mouse polyclonal antibody against NUPR1L was prepared by Genex Health Co., Ltd., and the specific antibodies to PFDN1 (ab151708), CD63 (ab59479), CD81 (ab79559), Integrin α6 (ab97760), Integrin β4 (ab133682) and β-actin (ab8226) were purchased from Abcam (Cambridge, UK).

### Cell viability assay

Cell viability was assessed indirectly using the CCK-8 assay. SW620 cells were seeded in a 96 well plate at a density of 3,000 cells/well. After 12 h, cells were treated with the indicated miRNAs or exosomes. Then, cells were treated with 100 µl of fresh medium containing 10% CCK-8 reagent (DoJinDo Laboratories, Japan) for 1 h at 37 ℃. The absorbance values of the wells at 450 nm were detected using an automatic spectrometer (Multimode Reader, PerkinElmer, USA). The above operations were repeated at 0, 1, 2, 3 and 4 days after cell treatment.

### Migration and invasion assays

SW620 cells were treated with miRNA mimics or different exosomes for 24 h. The treated cells of each group were digested with 0.25% trypsin, and after PBS washing, the cells were re-suspended with a serum-free culture medium to make a single-cell suspension, and the cells were adjusted to 2×10^5^/mL. Matrigel was diluted with serum-free medium, then added to the upper chamber and placed in an incubator at 37 ℃ for 1 hour. The prepared single-cell suspension was spread in the upper chamber of the Transwell with 5×10^3^ / well and the lower chamber was added with a medium containing 20% FBS. After incubation at 37 ℃ for 24 hours, the cells and fragments in the upper chamber were removed with cotton swabs, and the cells on the bottom surface were fixed by 4% paraformaldehyde and stained with 4', 6-diamidino-2-phenylindole (DAPI) (Thermo Fisher Science). After washing with PBS, the number of cells passing through the membrane was counted under an inverted microscope (IX71, Olympus, Japan), and the relative invasive ability of the cells was estimated. The cell migration ability of each group was detected without Matrigel matrix gel coating on the upper chamber filter membrane of Transwell, and the other steps were the same as the invasion test. Five fields were randomly selected for cell count in each experiment.

### Uptake of PKH26-labeled exosomes by recipient cells

Exosomes of 40 μg were incubated with lipophilic tracer PKH26 solution at 37 ℃ for 10min. Exosome Spin Columns (MW 3000) (Thermo Fisher Scientific) were used to remove excess PKH26. Cells were inoculated on 6-well plates (1×10^4^ cells/ well) and incubated for 24h. Pkh26-labeled exosomes (20 μg) were added to the recipient cell culture medium and incubated at 37 ℃ for 4h. Recipient cells were washed three times with PBS, fixed with 4% paraformaldehyde at room temperature for 10min, and permeated with 0.1%Triton X-100 for 5 min. The cells were then stained with DAPI and visualized with a fluorescence microscope (IX71, Olympus, Japan).

Integrin β4 blocking antibody (#MAB2059) was from Merck. PKH26-labeled exosomes were pre-incubated with Integrin β4 blocking antibody (10 μg/ml) for 1 hour, and then co-cultured with SW620 cells for 4 hours. Fluorescence microscopy was used to detect exosome uptake.

### Statistical analysis

All data were processed by SPSS21.0 software and expressed as mean ±SD, and Shapiro-Wilk (W) normality test was carried out. For non-normally distributed data, the nonparametric Wilcoxon signed-rank test was used to evaluate the statistical differences between groups. When the data were close to normal distribution, one-way ANOVA was used to compare multiple sets of data, and LSD method was used to analyze the multiple comparisons of means. *P* < 0.05 was considered to be statistically significant.

## Results

### Differential expression of miRNAs between human colonic epithelial cell line NCM460 and colon cancer cell line SW620 in the cells and in the exosomes

**Figure 1:**
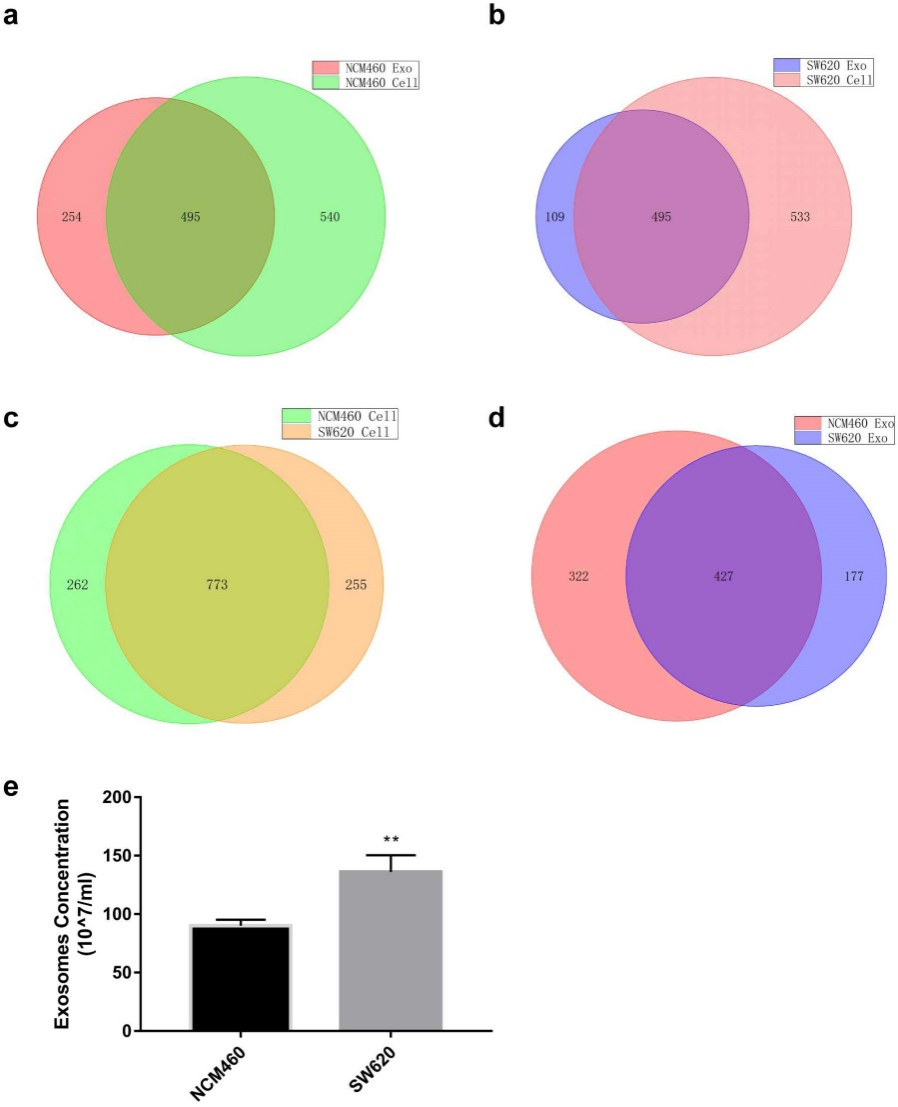
** Expression of miRNA in human normal colonic epithelial cell line NCM460 and colon cancer cell line SW620 as well as their exosomes.** (a) Venn diagram of intracellular and exosome miRNAs of NCM460 cells; (b) Venn diagram of intracellular and exosome miRNAs of SW620 cells; (c) Venn diagram of intracellular miRNAs of NCM460 and SW620 cells; (d) Venn diagram of exosome miRNAs of NCM460 and SW620 cells; (e) Abundance of exosomes secreted by SW620 and NCM460 cells. Data are the means ± SD. of three independent experiments. *P < 0.05.

miRNA expression profiles of human normal colonic epithelial cells NCM460 and colon cancer cells SW620 were examined by high-throughput sequencing, which yielded some interesting results (Tables S3 and S4). First, miRNA species in the exosomes were less abundant than in the cells for both cell lines (749 vs. 1035 for NCM460, 604 vs. 1028 for SW620) (Fig. 1a and b). No statistically significant difference was detected in intracellular miRNA species between NCM460 and SW620 cells (1035 vs. 1028, Fig. 1c), but the miRNA species in SW620 exosomes were much less abundant than in NCM460 exosomes (604 vs. 749, Fig. 1d). Then we examined the difference in number of exosomes secreted from NCM460 and SW620 cells. It was found that the number of exosomes secreted by SW620 cells was significantly greater than that by NCM460 cells (Fig. 1e), opposite to the abundance of miRNA species.

Such differences may make the SW620 cells more selective in exosome secretion, namely, larger number of miRNAs concentrated in a smaller set. We then analyzed the intracellular and exosome miRNAs of SW620 cells using NCM460 cells as control. According to the sequencing results, we defined 2 sets of differentially expressed miRNAs: “high in exosome and high in cell” (HEHC) and “high in exosome but low in cell” (HELC). The HEHC set included 39 miRNAs, including 19 passenger-strand miRNAs (48.71%) (Table S4); while the HELC set included 51 miRNAs, including 19 passenger-strand miRNAs (37.25%) (Table S5). These results strongly suggest that passenger-strand miRNAs are highly concentrated in exosomes.

Do these differentially expressed miRNAs have biological functions? According to current studies on exosomes, we preliminarily divided these miRNAs into two sets. One set that are actively released by tumor cells and perform specific functions, which are expected to be highly expressed in both tumor cells and exosomes compared to normal cells. And the other set that are detrimental to the tumor cells and are actively secreted by the tumor cells via exosomes, which are expected to be high in exosomes but low in tumor cells. Then, we searched the literature using “name of specific miRNA” and “tumor promotion”/ “tumor inhibition” as key words. The results suggested that most of the miRNAs in the HEHC set were carcinogenic (Table S5, Supplementary Reference), while most of the miRNAs in the HELC set were tumor-suppressing (Table S6, Supplementary Reference).

### Functional study of miR-2277-3p and miR-26b-3p in SW620 cells

**Figure 2:**
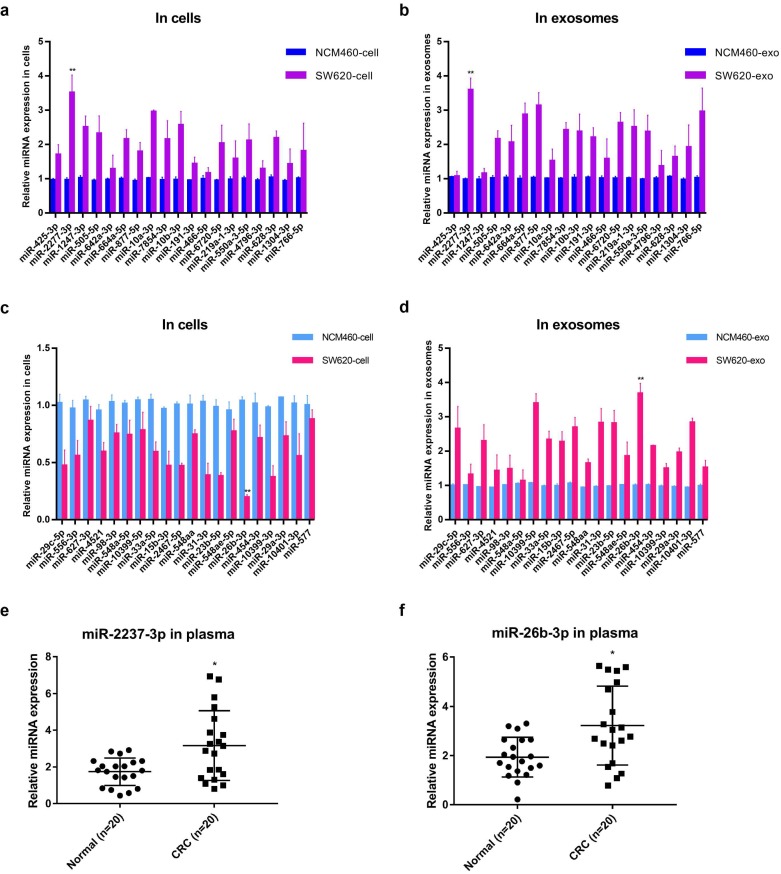
Quantitative PCR analysis of the relative expression levels of miRNA* in cells and exosomes in two sets (HEHC and HELC). (a, b) qRT-PCR assays were performed to compare the expression levels of miRNA* of HEHC set in NCM460 and SW620 cells and exosomes separately; (c, d) qRT-PCR assays were performed to compare the expression levels of miRNA* of HELC set in NCM460 and SW620 cells and exosomes separately. (e, f) qRT-PCR analysis of miR-2277-3p and miR-26b-3p expression in serum of colon cancer patients and healthy controls. U6 served as an internal control for intracellular miRNAs detection, and cel-mir-39 was used as the external control for miRNAs in exosomes. Data are the means ± SD. of three independent experiments. *P < 0.05 and ** P<0.01 versus control.

Further, the relative contents of miRNA* in cells and exosomes in the two sets were detected by quantitative PCR analysis, and the results were basically consistent with the sequencing results, among which miR-2277-3p (miR-2277*) and miR-26b-3p (miR-26b*) showed the most significant difference (Fig. 2a-d). Similar to the results obtained in cultured cells, the relative levels miR-2277-3p and miR-26b-3p in serum exosomes of colorectal cancer patients were also significantly higher than those of healthy controls (Fig. 2e, f). Based on the above hypothesis and results, miR-2277-3p was selected from the HEHC set and miR-26b-3p from the HELC set for further investigation. Both miR-2277-3p and miR-26b-3p are passenger-strand miRNAs, and few studies on their function in colorectal cancer have been reported.

**Figure 3:**
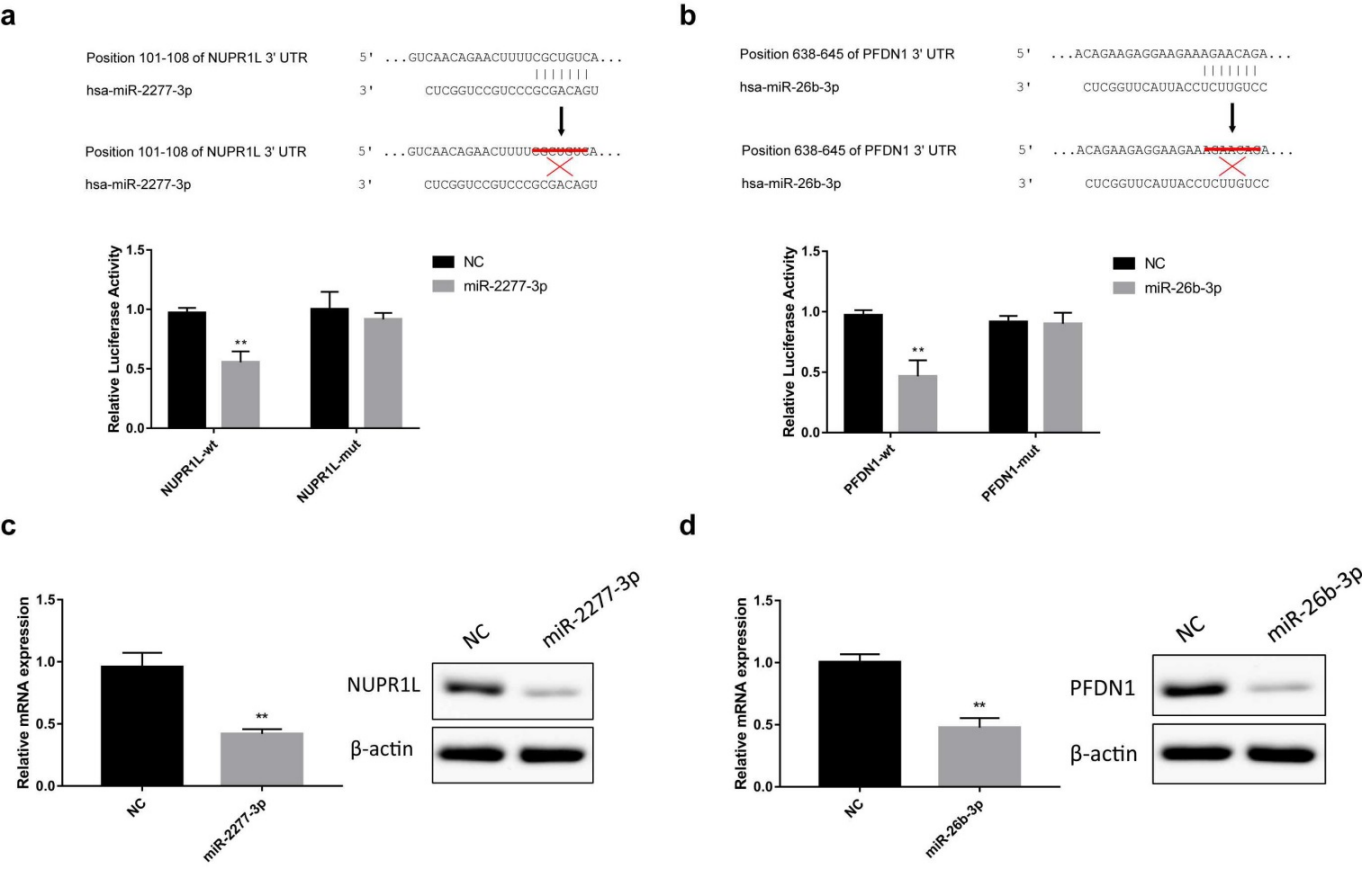
** Direct targeting of miR-2277-3p and miR-26b-3p on NUPR1L and PFDN1.** (a) Luciferase reporter vectors containing wild-type or mutated 3'-UTR of NUPR1L were constructed to test direct targeting of miR-2277-3p on NUPR1L mRNA; (b) Luciferase reporter vectors containing wild-type or mutated 3'-UTR of PFDN1 were constructed to test direct targeting of miR-26b-3p on PFDN1 mRNA; (c) The effect of miR-2277-3p on mRNA and protein expression of NUPR1L was examined by qPCR and Western blot; (d) The effect of miR-26b-3p on mRNA and protein expression of PFDN1 was examined by qPCR and Western blot. Data are the means ± SD. of three independent experiments. *P < 0.05 and ** *P*<0.01 versus control.

The online analysis tools miRWalk (http://mirwalk.umm.uni-heidelberg.de/), miranda (http://www.microrna.org/microrna/home.do) and Targetscan (http://www.Targetscan.org/vert_72/) predicted that NUPR1L and PFDN1 are potential targets of miR-2277-3p and miR-26b-3p, respectively. To verify this prediction, we constructed luciferase reporter vectors containing wild-type or mutant 3'-UTR sequences of NUPR1L or PFDN1 mRNAs. Luciferase activity assay showed that miR-2277-3p and miR-26b-3p significantly inhibited luciferase activity of the reporter vector containing the wild-type 3'-UTR but not the mutant 3'-UTR (Fig. 3a and b). In addition, after transfection of miR-2277-3p/miR-26b-3p mimics into SW620 cells, their targets NUPR1L/PFDN1 were significantly down-regulated at both mRNA and protein levels (Fig. 3c and d). These results demonstrated that NUPR1L and PFDN1 are direct targets of miR-2277-3p and miR-26B-3P, respectively.

**Figure 4:**
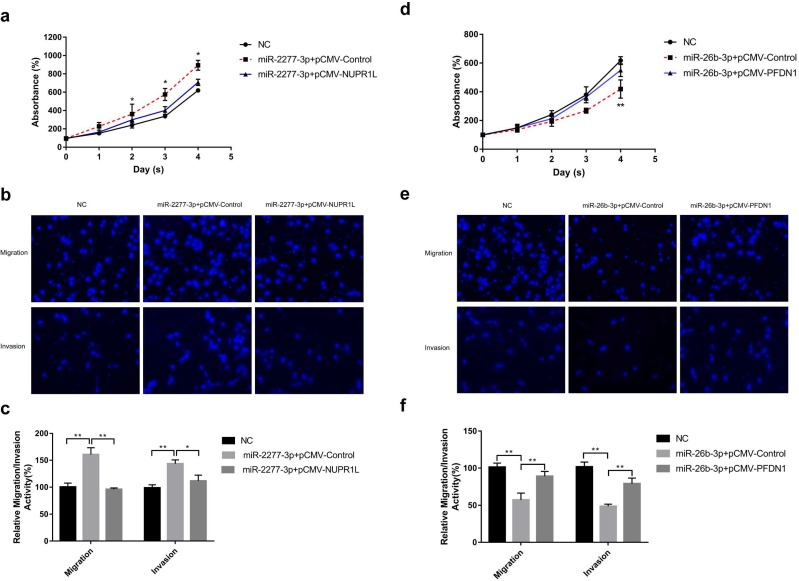
** The effect of miR-2277-3p and miR-26b-3p overexpression on proliferation, migration, and invasion of colon cancer cell line SW620.** (a) 20 nM miR-2277-3p was transfected into SW620 cells, and the proliferation activity of SW620 cells were determined by cck-8 assay at the given time points; (b, c) 24 hours after transfecting 20 nM of miR-2277-3p into the SW620 cells, cell migration and invasion were determined by transwell assay; (d) 20 nM miR-26b-3p was transfected into SW620 cells, and the proliferation activity of SW620 cells were determined by cck-8 assay at the given time points; (e, f) 24 hours after transfecting 20 nM of miR-26b-3p into the SW620 cells, cell migration and invasion were determined by transwell assay; Data are the means ± SD. of three independent experiments. *P < 0.05 and ** *P*<0.01 versus control.

Overexpression of miR-2277-3p in SW620 cells by liposome transfection resulted in increased cell proliferation, migration, and invasion (Fig. 4a and b), while overexpression of its target NUPR1L partially reversed its carcinogenic effect (Fig. 4a and b). In contrast, overexpressing miR-26b-3p inhibited proliferation, migration, and invasion of SW620 cells, while overexpression of its target NUPR1L partially reversed its tumor-suppressing effect (Fig. 4c and d).

### Paracrine effect of exosomes rich in miR-2277-3p or miR-26b-3p

**Figure 5:**
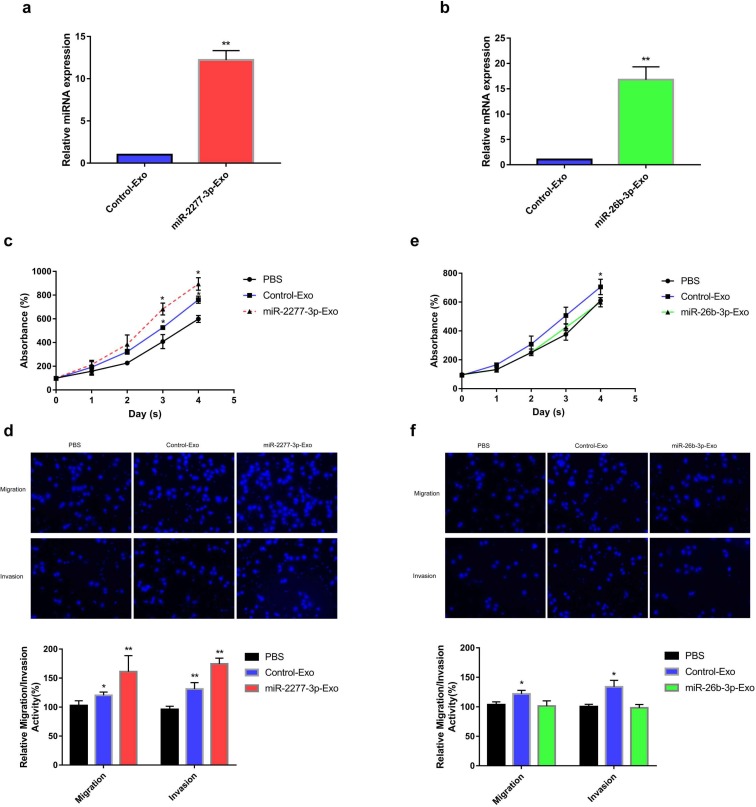
** Paracrine effect of exosomes rich in miR-2277-3p or miR-26b-3p.** (a) 12 hours after transfecting 100 μM miR-2277-3p into SW620 cells, the supernatant was collected and purified to prepare exosomes rich in miR-2277-3p. miR-2277-3p content in exosomes from the transfected cells and control cells were determined by qPCR. Data were normalized to exogenous cel-miR-39; (b) 12 hours after transfecting 100 μM miR-26b-3p into SW620 cells, the supernatant was collected and purified to prepare exosomes rich in miR-26b-3p. miR-26b-3p content in exosomes from the transfected cells and control cells were determined by qPCR; (c) The effect of miR-2277-3p-Exo on proliferation of untransfected SW620 cells; (d) The effect of miR-2277-3p-Exo on migration and invasion of untransfected SW620 cells; (e) The effect of miR-26b-3p-Exo on proliferation of untransfected SW620 cells; (f) The effect of miR-26b-3p-Exo on migration and invasion of untransfected SW620 cells. Data are the means ± SD. of three independent experiments. *P < 0.05 and ** P<0.01 versus control.

**Figure 6:**
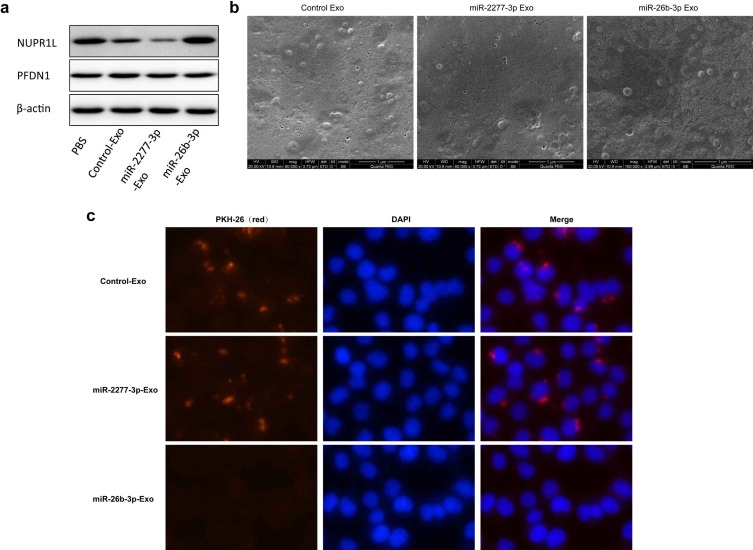
** Uptake of miR-2277-3p-Exo and miR-26b-3p-Exo up by recipient cells.** (a) After 24 hours of incubation with exosomes of each group, the untransfected SW620 cells were examined by Western blot to determine the effects of miR-2277-3p and miR-26b-3p on protein expression of their targets; (b) Transmission electron microscopy of exosomes of each group isolated by the ExoQuick-TC kit.; (c) By PKH-26 labeling and fluorescence microscopy to examine uptake of the exosomes by untransfected SW620 cells during 4 hours of co-incubation.

Given that overexpression of miR-2277-3p or miR-26b-3p in SW620 cells showed significant biological function, we were curious whether exosomes containing these miRNAs show similar paracrine effects. So, we overexpressed miR-2277-3p or miR-26b-3p in SW620 cells, collected exosomes rich in miR-2277-3p or miR-26b-3p, and incubated these exosomes with untransfected SW620 cells to see their paracrine effects. The results showed that after overexpressing miR-2277-3p or miR-26b-3p in the SW620 cells, relative content of the corresponding miRNA significantly increased in the exosomes (Fig. 5a and b), which were then named miR-2277-3p-Exo and miR-26b-3p-Exo, respectively. Co-incubation with miR-2277-3p-Exo significantly promoted proliferation, migration, and invasion of the tumor cells (Fig. 5c and d), similar to that caused by miR-2277-3p overexpression; however, unlike miR-26b-3p overexpression, miR-26b-3p-Exo showed no significant effect on proliferation, migration, and invasion of the tumor cells (Fig. 5e and f). Then we examined the effect of miR-2277-3p-Exo and miR-26b-3p-Exo on their miRNA targets, the results demonstrated that miR-2277-3p-Exo significantly downregulated NUPR1L expression, while miR-26b-3p-Exo failed to downregulate PFDN1 expression (Fig. 6a). What is the difference between the two types of exosomes? Electron microscopy showed no significant difference in morphology between miR-2277-3p-Exo and miR-26b-3p-Exo (Fig. 6b), but fluorescence-labeling revealed that uptake of miR-26b-3p-Exo by the target cells was significantly lower than that of miR-2277-3p-Exo (Fig. 6c). These results suggested that, although we successfully prepared exosomes rich in either miR-2277-3p or miR-26b-3p, their paracrine effects are not guaranteed since the uptake of the exosomes can vary greatly.

### Key role of integrin β4 in the uptake of exosomes by colon cancer cells

**Figure 7:**
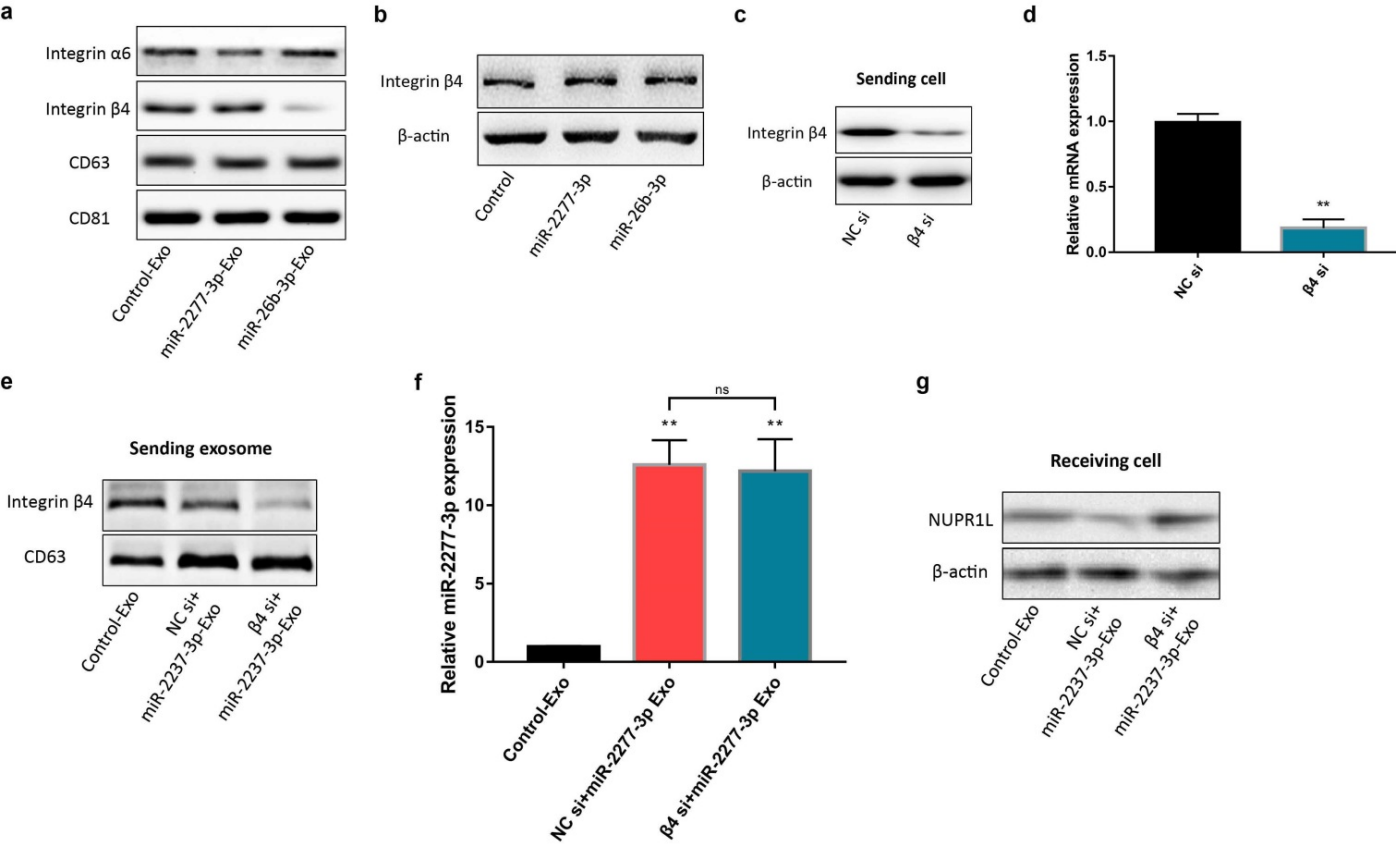
** miR-2277-3p-Exo and miR-26b-3p-Exo from SW620 cells showed different expression levels of integrins.** (a) Content of integrin α6 and β4 in miR-2277-3p-Exo and miR-26b-3p-Exo determined by Western blot; (b) Content of integrin β4 in the source cells of Control-Exo, miR-2277-3p-Exo and miR-26b-3p-Exo were examined by Western blot. (c, d) mRNA and protein levels of integrin β4 in each group of SW620 cells 24 hours after transfection with integrin β4-siRNA; (e) The SW620 cells were co-transfected with negative control (NC) si or integrin β4-siRNA (10 nM) and miR-2277-3p (100nM), then exosomes were collected from supernatant 24 hours after transfection, and β4 protein expression examined by Western blot with CD63 as control; (f) MiR-2277-3p content in exosomes was measured by qPCR. Data were normalized to exogenous cel-miR-39; (g) The exosomes were incubated with recipient SW620 cells, and NUPR1L expression in these cells were determined by Western blot. Data are the means ± SD. of three independent experiments. *P < 0.05, ** P<0.01 versus control, and ns= nonsignificant.

Previous studies have shown that primary tumors may release a large number of exosomes that fertilize subsequent metastasis. In addition, the exosomes are also decorated with integrin receptors that direct transportation of the exosomes to specific organs and tissues like zip codes 12. In other words, integrins on the surface of exosomes may play a crucial role in the targeted transport and delivery of exosomes. Integrin α6 and β4 are the specific integrins rich in the exosomes from colon cancer cells 24. We compared the content of integrins α6 and β4 on Control-Exo, miR-2277-3p-Exo, and miR-26b-3p-Exo. The results demonstrated no significant difference in integrin α6 content among the three types of exosomes, while integrin β4 content was significantly lower in miR-26b-3p-Exo than the other two types of exosomes (Fig. 7a), suggesting that integrin β4 may contribute to the uptake of the exosome by the recipient cells.

**Figure 8:**
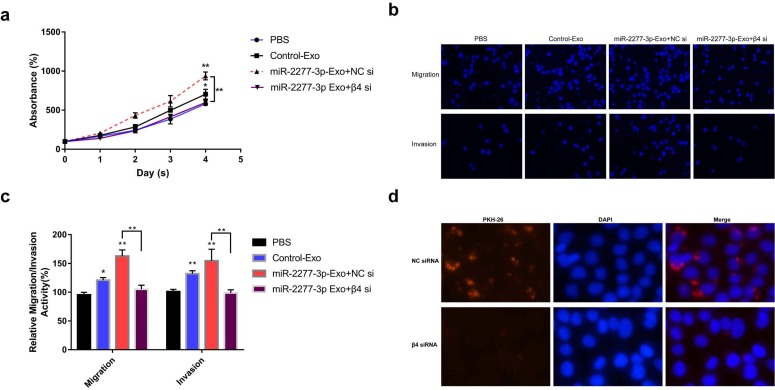
** Integrin β4 knock-down attenuated the paracrine effect of miR-2277-3p-Exo.** (a) Proliferation of recipient SW620 cells when incubated with miR-2277-3p-Exo produced by integrin β4-silenced or control cells; (b, c) Migration and invasion of recipient SW620 cells when incubated with miR-2277-3p-Exo produced by integrin β4-silenced or control cells; (d) PKH-26 labeling and immunocytochemistry to observe uptake of the miR-2277-3p-Exo produced by integrin β4-silenced or control cells after 4 hours of incubation. Data are the means ± SD. of three independent experiments. *P < 0.05 and ** P<0.01 versus control.

**Figure 9:**
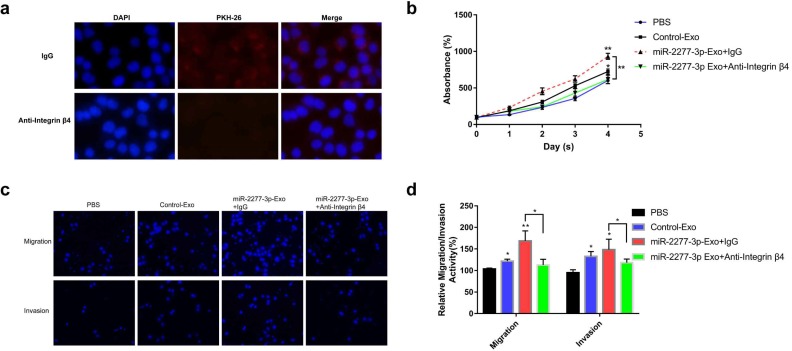
** Integrin β4-specific antibody effectively blocked the paracrine effect of miR-2277-3p-Exo.** (a) Each group of labeled exosomes was pretreated with integrin β4-specific antibody (10 μg/ml) or control IgG for 1 hour before incubation with SW620 cells for 4 hours, and uptake of the exosomes in each group were observed under a fluorescence microscope; (b) Pretreatment with anti-integrin β4 antibody significantly attenuated the effect of miR-2277-3p-Exo to promote cell proliferation; (c, d) Pretreatment with anti-integrin β4 antibody significantly attenuated the effect of miR-2277-3p-Exo to promote cell migration and invasion. Data are the means ± SD. of three independent experiments. *P < 0.05 and ** P<0.01 versus control.

**Figure 10:**
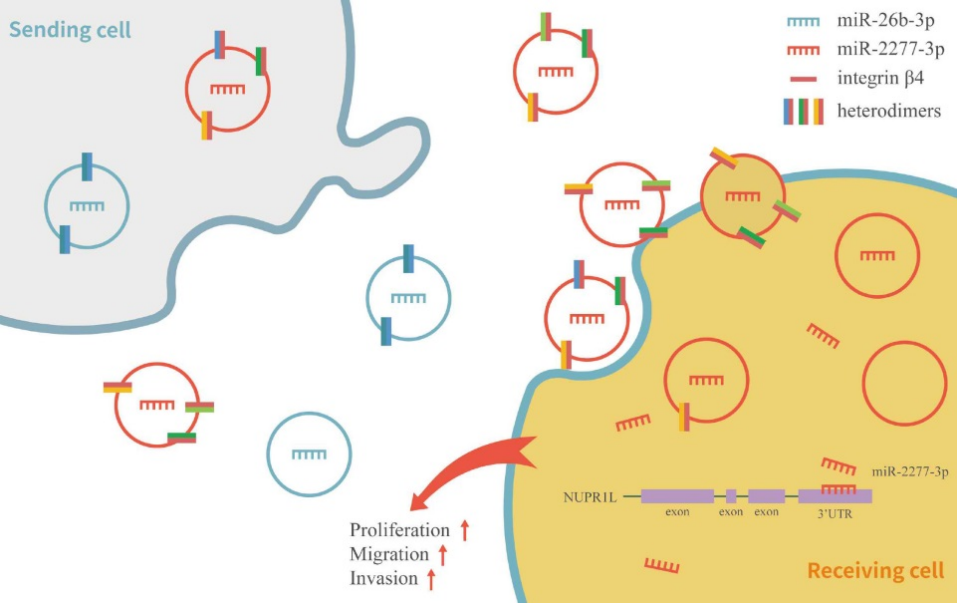
Schematic model of integrin β4 regulating the heterogeneity of exosomes function in SW620 cells.

To verify this assumption, we first need to know whether the overexpression of the two miRNAs* leads to the difference of integrin β4 in cells, and ultimately affects the content of integrin β4 in exosomes. We compared the relative amounts of integrin β4 in the source cells of Control-Exo, miR-2277-3p-Exo, and miR-26b-3p-Exo. The results showed that there was no significant difference in integrin β4 among SW620 cells in three states (Fig. 7b). Next, we knocked down integrin β4 expression when preparing miR-2277-3p-Exo (Fig. 7c and d), which had no effect on miR-2277-3p content in the exosomes (Fig. 7f) but significantly decreased integrin β4 content (Fig. 7e). Co-incubation of the SW620 recipient cells with these exosomes showed no significant effect on the expression of NUPR1L, the target of miR-2277-3p (Fig. 7g). It can be concluded that integrin β4 knockdown significantly attenuated the effect of miR-2277-3p-Exo to promote proliferation (Fig. 8a), migration, and invasion of recipient cells (Fig. 8b, c). In addition, fluorescence microscopy results demonstrated that integrin β4 knockdown prevented uptake of the exosomes by the recipient cells (Fig. 8d). Similarly, if integrin β4 was blocked with specific antibody during co-incubation of the exosomes with the recipient cells, the results would be similar to that after integrin β4 knock-down - miR-2277-3p-Exo was not effectively uptaken (Fig. 9a), and its function to promote proliferation, migration, and invasion of recipient cells was attenuated (Fig. 9b-d). These results strongly suggest that integrin β4 plays a pivotal role in exosome uptake by SW620 colon cancer cells (Fig. 10).

## Discussion

Although miRNAs have been extensively studied, the functions of passenger-strand miRNAs have rarely been studied so far. Some scholars believe that the main role of passenger-strand miRNAs is to influence RISC assembly. During the assembly of RISC, blockage of degradation or dissociation of the passenger strand may impair the assembly of RISC, and thus, attenuate the silencing effect of the guide-strand miRNA 25. In recent years, some scholars have discovered that the passenger-strand miRNAs not only affect the assembly of RISC but also are integrated into RISC to play the same gene-silencing role as the guide strand. For example, miR-30e* promotes invasion of glioma cells by acting on IκBα 26; miR-378* mediates metabolic conversion of breast cancer cells by targeting the PGC-1β/ERRγ pathway 27; miR-29c* inhibits progression of gallbladder cancer by directly targeting CPEB4 and thus inhibiting the MAPK pathway 28, and miR-199a* controls expression of the proto-oncogene MET 29. It seems that miRNA* silencing is ubiquitous in a variety of cells and plays an important role. However, most miRNA*s are still overlooked due to their relatively low level compared to the guide strand. Bang et al. demonstrated, for the first time, that cardiac fibroblasts can release large amounts of miRNA* into exosomes to communicate with cardiomyocytes 21. In this study, we demonstrated that human colon cancer cell line SW620 is also capable of secreting a large number of passenger-strand miRNAs in exosomes (Tables S1 and S2). It should be noted that high abundance of passenger-strand miRNAs in exosomes may be a cause of their low level inside the cells.

To address this issue, we proposed the hypothesis that miRNAs may be actively or passively released: tumor cells may express a high level of miRNAs that are beneficial to their growth, which are then passively enriched in the exosomes, or tumor cells may tend to expel miRNAs that are detrimental to their growth, so that these miRNAs may have high level in the exosomes but low level inside the cells. Based on these assumptions, we divided the differentially expressed miRNAs into two sets, those “high in exosome and high in cell” and those “high in exosome but low in cell.” Literature studies suggested that miRNAs “high in exosome and high in cell” are mostly carcinogenic, while miRNAs “high in exosome but low in cell” show considerable anti-cancer effects (Supplementary Reference). Such classification may seem relatively preliminary, but it still could reflect the heterogeneity of exosome miRNAs secreted by the same tumor cells. MiR-2277-3p and miR-26b-3p were selected as representatives from the two categories. These two miRNAs were overexpressed in colon cancer cells via liposome transfection, and as expected, miR-2277-3p promoted cancer by targeting NUPR1L, while miR-26b-3p acted as a tumor suppressor by targeting PFDN1. Two functionally distinct miRNAs were secreted via exosomes by the same group of cells, but would they exert contrary paracrine effect on the recipient cells? To test this we prepared exosomes rich in miR-2277-3p and miR-26b-3p by overexpression and found that miR-2277-3p-Exo co-incubation exerted a similar influence as miR-2277-3p overexpression, while miR-26b-3p-Exo co-incubation failed to reflect the effect of miR-26b-3p overexpression. Further experiments revealed a huge difference in the ability of the two types of exosomes to be uptaken by recipient cells, in which integrin β4 was proved to play a key role. These results suggest that colon cancer cells may have a specific mechanism to classify and label different miRNAs during their package and release, to maximize their benefits and minimize their harm. In other words, exosomes secreted by the same group of cells may be heterogenic.

In this study, we did not dig further into the differences in other contents of miR-2277-3p-Exo and miR-26b-3p-Exo, such as differences in proteins, RNAs, and lipids. We were not sure whether such artificially prepared exosomes are consistent with those naturally secreted by tumor cells. To address this issue, we had intended to isolate the naturally occurring exosomes using magnetic beads conjugated with anti-integrin β4 antibody, which, unfortunately, was not commercially available at present. In addition, this study revealed the role of integrin β4 preliminarily. Yet, integrins are heterodimers composed of an alpha subunit and a beta subunit via non-covalent linkage, and with at least 18 alpha subunits and eight beta subunits currently identified, there are over 20 possible combinations 30, 31. More detailed research in the future is required to explore in-depth in multiple cells and cancers.

## Conclusions

We revealed the fact that a large number of passenger-strand miRNAs exist in exosomes of colon cancer cells, these miRNAs are preliminarily categorized into two sets, and miR-2277-3p and miR-26b-3p, as representatives of each set, showed opposite functions. In addition, we revealed that integrin β4 is a marker of exosome heterogeneity in colon cancer cells, which directly correlates with the ability of exosomes to be uptaken by cells of the same kind, thus regulating the paracrine effect of exosomes.

## Supplementary Material

Supplementary table 1, 2, 4, 5.

Supplementary table 3.

Supplementary table 4.

## Abbreviations

miRNA: microRNA
miRNA*: passenger strand miRNA
HEHC: high in exosome and high in cell
HELC: high in exosome but low in cell
NUPR1L: Nuclear Transcriptional Regulator 1-Like Protein
PFDN1: Prefoldin Subunit 1
QRT-PCR: quantitative real-time polymerase chain reaction
TEM: Transmission electron microscopy
