# Supplementary material for: Functional Passenger-Strand miRNAs in Exosomes Derived from Human Colon Cancer Cells and Their Heterogeneous Paracrine Effects
Source: Int J Biol Sci. 2020 Feb 4;16(6):1044–58. doi: 10.7150/ijbs.40787 (PMC7053335; doi:10.7150/ijbs.40787)
Supplement: Supplementary file 1 — Supplementary table 1, 2, 4, 5. [file ijbsv16p1044s1.pdf]

Table S1. Patients Characteristics

**Characteristics**	Colorectal cancer (n=20)
**Gender**	
Male	12
Female	8
**Age at diagnosis**	
Mean±SD	60±12
Median (range)	56 (32-81)
**Clinical stage**	
I	4
II	11
III	5
**Nodal status**	
Positive	12
Negative	8
**Tumor location**	
Rectum	10
Distal colon	4
Proximal colon	6
**Histology**	
Adenocarcinoma	15
Mucous adenocarcinoma	5
signet-ring cell carcinoma	0

Supplementary Table S2: Primers for miRNA* RT-qPCR

miR_name	Forward primer	Reverse primer
hsa-miR-425-3p	gcatcgggaatgtcgtgt	agttttttttttttttttggcgga
hsa-miR-2277-3p	cagcgccctgcct	agttttttttttttttttcgagcca
hsa-miR-1247-3p	cgggaacgtcgagac	ccagttttttttttttttttgctcca
hsa-miR-505-5p	ggggagccaggaagt	gtccagttttttttttttttttaacatca
hsa-miR-642a-3p	gcagagacacatttggagag	ggtccagttttttttttttttttgttc
hsa-miR-766-5p	cagaggaggaattggtgct	ggtccagttttttttttttttttagac
hsa-miR-664a-5p	cagactggctagggaaaatg	gtccagttttttttttttttttccaatc
hsa-miR-877-5p	gtagaggagatggcgca	ggtccagttttttttttttttttgtc
hsa-miR-10a-3p	gcgcagcaaattcgtatc	ggtccagttttttttttttttttattcc
hsa-miR-7854-3p	gaggtgaccgcagatg	ggtccagttttttttttttttttcttc
hsa-miR-10b-3p	cgcagcagattcgattc	ggtccagttttttttttttttttattcc
hsa-miR-191-3p	ctgcgcttggatttcgt	ccagttttttttttttttttgggga
hsa-miR-466-5p	cagtgtgttgcatgtgtg	ggtccagttttttttttttttttacatatac
hsa-miR-6720-5p	agttccagccctggtag	cagttttttttttttttttacgcgg
hsa-miR-219a-1-3p	gcagagagttgagtctggac	ccagttttttttttttttttacggga
hsa-miR-550a-3-5p	gagtgcctgagggagt	ggtccagttttttttttttttttctct
hsa-miR-4796-3p	cgcagtaaagtggcagagt	ggtccagttttttttttttttttgtc
hsa-miR-628-3p	gcagttctagtaagagtggca	gtccagttttttttttttttttcgac
hsa-miR-1304-3p	gcagtctcactgtagcatc	cagttttttttttttttttggggttc
hsa-miR-29c-5p	accgatttctcctggtgt	ggtccagttttttttttttttttctga
hsa-miR-556-3p	gcgcagatattaccattagctc	ggtccagttttttttttttttttaagatg
hsa-miR-627-3p	gcagctcttttctttgagactc	ggtccagttttttttttttttttagtg
hsa-miR-98-3p	gcgcagctatacaacttactac	ggtccagttttttttttttttttagga
hsa-miR-548a-5p	gcagaaaagtaattgcggtct	ggtccagttttttttttttttttacca
hsa-miR-10399-5p	gcgcagaattacagattgtct	gtccagttttttttttttttttctctga
hsa-miR-33a-5p	cgcaggtgcattgtagt	gtccagttttttttttttttttgcaat
hsa-miR-15b-3p	cgcagcgaatcattatttgc	aggtccagttttttttttttttttagag
hsa-miR-2467-5p	gtgaggctctgttagcctt	ccagttttttttttttttttagagcca
hsa-miR-548aa	cgcagaaaaccacaattacttttg	gtccagttttttttttttttttgtgc
hsa-miR-31-3p	gtgctatgccaacatattgc	ggtccagttttttttttttttttgatg
hsa-miR-23b-5p	gggttcctggcatgct	caggtccagttttttttttttttttaaatc
hsa-miR-548ae-5p	cgcagaaaagtaattgtggt	tccagttttttttttttttttggca
hsa-miR-26b-3p	cagcctgttctccattacttg	gtccagttttttttttttttttgagc
hsa-miR-454-3p	cgcagtagtgcaatattgct	gtccagttttttttttttttttaaccct
hsa-miR-10399-3p	gtctctcggacaagctgt	ggtccagttttttttttttttttaacct
hsa-miR-29a-3p	cgcagtagcaccatctga	tccagttttttttttttttttaaccga
hsa-miR-10401-3p	aggacctcgccgtc	gtccagttttttttttttttttggc
hsa-miR-577	gcgcaggtagataaaatattggt	ggtccagttttttttttttttttcag

Supplementary Table S4：miRNAs set of higher level in the exosomes and higher level inside the cells (SW620 vs. NCM460, Exo|Cell).

High in exosome and high in cell (HEHC)											
miR_name	miR_seq	up/down	fold_change(SW_620/NCM_460)(Exo|Cell)	log2(fold_change)	pvalue(chi_square_2x2)	pvalue(fisher_test)	NCM_460(norm)	SW_620(norm)	NCM_460 (raw)	SW_620(raw)	Expression level
hsa-miR-425-3p_L+1R-1[81] *	CATCGGGAATGTCGTGTCCGCC	up|up	2.82|3.20	1.49|1.68	1.25E-74|1.52E-76	2.57E-76|1.20E-78	280|213	789|682	72|392	509|1,505	middle|middle
hsa-miR-2277-3p_R+1 *	TGACAGCGCCCTGCCTGGCTCG	up|up	inf|11.72	inf|3.55	1.95E-05|9.25E-38	3.95E-06|7.81E-42	0|15	16|172	0|27	10|380	middle|middle
hsa-miR-1247-3p_L-3[82] *	CGGGAACGTCGAGACTGGAGC	up|up	inf|2.65	inf|1.41	2.89E-06|1.06E-02	3.83E-07|1.64E-02	0|6	19|16	0|11	12|35	middle|middle
hsa-miR-505-5p_R+1[83] *	GGGAGCCAGGAAGTATTGATGTT	up|up	inf|4.95	inf|2.31	5.25E-18|1.75E-08	2.44E-22|5.97E-09	0|9	64|46	0|17	41|101	middle|middle
hsa-miR-1343-3p	CTCCTGGGGCCCGCACTCTCGC	up|up	inf|2.41	inf|1.27	2.53E-03|2.43E-08	1.99E-03|2.04E-08	0|35	8|85	0|65	5|188	middle|middle
hsa-miR-628-5p[84, 85]	ATGCTGACATATTTACTAGAGG	up|up	inf|2.04	inf|1.03	6.58E-08|2.20E-06	3.61E-09|2.80E-06	0|39	25|79	0|71	16|174	middle|middle
hsa-miR-642a-3p_R-1[86] *	AGACACATTTGGAGAGGGAAC	up|up	inf|inf	inf|inf	5.25E-18|1.38E-12	2.44E-22|3.85E-15	0|0	64|42	0|0	41|92	middle|middle
hsa-miR-766-5p_R-1[87] *	AGGAGGAATTGGTGCTGGTCT	up|up	inf|inf	inf|inf	3.53E-04|1.95E-02	1.93E-04|1.92E-02	0|0	11|5	0|0	7|10	middle|middle
hsa-miR-664a-5p_R-2[88] *	ACTGGCTAGGGAAAATGATTGG	up|up	inf|inf	inf|inf	1.35E-15|1.69E-06	5.81E-19|3.01E-07	0|0	54|19	0|0	35|42	middle|middle
hsa-miR-877-5p_R+3[89] *	GTAGAGGAGATGGCGCAGGGGAC	up|up	inf|2.59	inf|1.37	4.17E-53|7.54E-26	2.87E-68|6.56E-26	0|107	200|276	0|196	129|609	middle|middle
hsa-miR-10a-3p_R-1	CAAATTCGTATCTAGGG	up|up	3.44|2.97	1.78|1.57	0.00E+00|6.06E-	4.47E-274|3.5	677|198	2,332|589	174|364	1,504|1,3	middle|mi

*	GAAT				62	6E-63				00	ddle
hsa-mir-4454-p5	TCCGAGTCACGGCACCAAA	up|up	7.45|66.37	2.90|6.05	0.00E+00|0.00E+00	3.51E-267|0.00E+00	183|111	1,363|7,335	47|203	879|16,180	high|high
hsa-miR-361-5p[90-102]	TTATCAGAATCTCCAGGGGTAC	up|up	2.69|2.02	1.43|1.01	0.00E+00|0.00E+00	0.00E+00|0.00E+00	2,771|3,505	7,457|7,076	712|6,438	4,809|15,609	high|high
hsa-miR-7854-3p_R+1 *	TGAGGTGACCGCAGATGGGAAG	up|up	inf|inf	inf|inf	5.25E-18|5.20E-05	2.44E-22|1.57E-05	0|0	64|14	0|0	41|30	middle|middle
hsa-miR-98-5p[103-105]	TGAGGTAGTAAGTTGTATTGTT	up|up	6.28|2.04	2.65|1.03	0.00E+00|0.00E+00	0.00E+00|0.00E+00	599|4,226	3,764|8,633	154|7,762	2,427|19,043	high|high
hsa-miR-10b-3p_L-1[106, 107] *	CAGATTCGATTCTAGGGGAAT	up|up	inf|inf	inf|inf	1.12E-06|1.05E-08	1.76E-07|5.42E-10	0|0	20|27	0|0	13|60	middle|middle
hsa-miR-425-5p[108-115]	AATGACACGATCACTCCCGTTGA	up|up	2.67|2.50	1.42|1.32	0.00E+00|0.00E+00	9.89E-146|0.00E+00	611|2,181	1,630|5,443	157|4,006	1,051|12,007	high|high
hsa-miR-466_R-1_1ss22AC[116-119]	ATACACATACACGCAACACACC	up|up	inf|inf	inf|inf	1.95E-05|9.30E-06	3.95E-06|3.23E-06	0|0	16|16	0|0	10|36	middle|middle
hsa-miR-191-3p_L-1 *	CTGCGCTTGGATTTCGTCCCC	up|up	inf|2.44	inf|1.29	1.34E-04|8.62E-03	8.86E-05|1.11E-02	0|8	12|19	0|14	8|41	middle|middle
hsa-mir-466-p5 *	TGTGTTGCATGTGTGTATATGT	up|up	inf|inf	inf|inf	2.06E-24|1.72E-03	1.91E-30|1.80E-03	0|0	88|8	0|0	57|18	middle|middle
hsa-miR-664a-3p[120]	TATTCATTTATCCCCAGCCTACA	up|up	inf|3.03	inf|1.60	2.57E-08|4.86E-24	1.66E-09|2.36E-24	0|71	26|214	0|130	17|473	middle|middle
hsa-miR-4286_R+1[121]	ACCCCACTCCTGGTACCA	up|up	inf|2.68	inf|1.42	3.34E-17|2.42E-35	5.47E-21|8.48E-36	0|137	60|368	0|252	39|812	middle|middle
hsa-miR-6720-5p_R+1 *	TTCCAGCCCTGGTAGGCGCCGCGT	up|up	inf|3.26	inf|1.71	9.39E-04|9.59E-04	9.13E-04|1.95E-03	0|7	9|21	0|12	6|47	middle|middle
hsa-miR-196a-5p[36, 122]	TAGGTAGTTTCATGTTG	up|up	5.35|2.99	2.42|1.58	0.00E+00|0.00E	0.00E+00|0.00	699|1,823	3,734|5,44	180|3,349	2,408|12,	high|high

<table>
<tr><td></td><td>TTGGG</td><td></td><td></td><td></td><td>+00</td><td>E+00</td><td></td><td>8</td><td></td><td>017</td><td></td></tr>
<tr><td>hsa-miR-219a-1-3p_R+1 *</td><td>AGAGTTGAGTCTGGACGTCCCGT</td><td>up|up</td><td>inf|2.68</td><td>inf|1.42</td><td>9.39E-04|1.91E-02</td><td>9.13E-04|3.08E-02</td><td>0|5</td><td>9|13</td><td>0|9</td><td>6|29</td><td>middle|middle</td></tr>
<tr><td>hsa-miR-4659a-3p</td><td>TTTCTTCTTAGACATGGCAACG</td><td>up|up</td><td>inf|3.61</td><td>inf|1.85</td><td>3.53E-04|1.03E-02</td><td>1.93E-04|8.41E-03</td><td>0|3</td><td>11|12</td><td>0|6</td><td>7|26</td><td>middle|middle</td></tr>
<tr><td>hsa-miR-550a-3-5p_R+1[123] *</td><td>AGTGCCTGAGGGAGTAAGAGA</td><td>up|up</td><td>inf|inf</td><td>inf|inf</td><td>2.11E-09|2.32E-03</td><td>7.39E-11|1.80E-03</td><td>0|0</td><td>30|8</td><td>0|0</td><td>20|17</td><td>middle|middle</td></tr>
<tr><td>hsa-miR-212-3p[124, 125]</td><td>TAACAGTCTCCAGTCACGGCC</td><td>up|up</td><td>inf|3.59</td><td>inf|1.84</td><td>7.49E-06|3.63E-09</td><td>1.82E-06|1.37E-09</td><td>0|17</td><td>17|63</td><td>0|32</td><td>11|138</td><td>middle|middle</td></tr>
<tr><td>hsa-miR-130b-3p[126]</td><td>CAGTGCAATGATGAAAGGGCAT</td><td>up|up</td><td>2.40|3.12</td><td>1.26|1.64</td><td>4.46E-85|4.28E-55</td><td>2.64E-86|3.43E-56</td><td>459|160</td><td>1,103|498</td><td>118|293</td><td>711|1,098</td><td>middle|middle</td></tr>
<tr><td>hsa-let-7i-5p[127]</td><td>TGAGGTAGTAGTTTGTGCTGTT</td><td>up|up</td><td>2.95|2.00</td><td>1.56|1.00</td><td>0.00E+00|0.00E+00</td><td>0.00E+00|0.00E+00</td><td>13,624|15,844</td><td>40,214|31,705</td><td>3,501|29,101</td><td>25,933|69,935</td><td>high|high</td></tr>
<tr><td>hsa-miR-4796-3p_R-1 *</td><td>TAAAGTGGCAGAGTATAGACA</td><td>up|up</td><td>inf|inf</td><td>inf|inf</td><td>3.53E-04|2.95E-04</td><td>1.93E-04|1.68E-04</td><td>0|0</td><td>11|11</td><td>0|0</td><td>7|24</td><td>middle|middle</td></tr>
<tr><td>hsa-miR-6720-3p</td><td>CGCGCCTGCAGGAACTGGTAGA</td><td>up|up</td><td>inf|14.60</td><td>inf|3.87</td><td>1.12E-06|8.15E-28</td><td>1.76E-07|2.10E-31</td><td>0|8</td><td>20|119</td><td>0|15</td><td>13|263</td><td>middle|middle</td></tr>
<tr><td>hsa-mir-1304-p5</td><td>CGGTTTGAGGCTACAGTGAGAT</td><td>up|up</td><td>4.02|5.71</td><td>2.01|2.51</td><td>1.00E-23|6.25E-50</td><td>1.69E-24|4.76E-53</td><td>43|52</td><td>172|295</td><td>11|95</td><td>111|651</td><td>middle|middle</td></tr>
<tr><td>hsa-miR-132-3p[128]</td><td>TAACAGTCTACAGCCATGGTCG</td><td>up|up</td><td>2.44|2.16</td><td>1.28|1.11</td><td>1.70E-67|2.42E-41</td><td>2.08E-68|1.75E-41</td><td>350|268</td><td>853|580</td><td>90|492</td><td>550|1,279</td><td>middle|middle</td></tr>
<tr><td>hsa-miR-1247-5p[129-131]</td><td>ACCCGTCCCGTTCGTCCCCGGA</td><td>up|up</td><td>inf|3.78</td><td>inf|1.92</td><td>0.00E+00|1.79E-55</td><td>2.56E-276|1.71E-57</td><td>0|112</td><td>816|424</td><td>0|206</td><td>526|936</td><td>middle|middle</td></tr>
<tr><td>hsa-miR-628-3p_L+1[132, 133] *</td><td>TTCTAGTAAGAGTGGCAGTCGA</td><td>up|up</td><td>2.19|2.67</td><td>1.13|1.42</td><td>8.32E-05|6.97E-26</td><td>9.21E-05|4.49E-26</td><td>23|100</td><td>51|266</td><td>6|183</td><td>33|587</td><td>middle|middle</td></tr>
<tr><td>hsa-miR-190b-5p</td><td>TGATATGTTTGATATTGG</td><td>up|up</td><td>inf|inf</td><td>inf|inf</td><td>5.16E-24|5.42E-</td><td>4.17E-30|6.21</td><td>0|0</td><td>87|21</td><td>0|0</td><td>56|46</td><td>middle|mi</td></tr>
</table>

	GTTG				07	E-08					ddle
hsa-miR-1304-3p_1ss13CA *	TCTCACTGTAGCATCGAACCCC	up|up	2.46|3.05	1.30|1.61	6.63E-28|7.51E-79	3.26E-28|9.31E-81	136|242	335|737	35|444	216|1,625	middle|middle
hsa-miR-504-5p	AGACCCTGGTCTGCACTCTATC	up|up	inf|inf	inf|inf	2.89E-06|6.36E-10	3.83E-07|1.04E-11	0|0	19|32	0|0	12|70	middle|middle

*: passenger strand

Supplementary Table S5: miRNAs set of higher level in the exosomes but lower level inside the cells (SW620 vs. NCM460, Exo|Cell).

High in exosome but low in cell (HELC)											
miR_name	miR_seq	up/down	fold_change(SW_620/NCM_460)(Exo|Cell)	log2(fold_change)	pvalue(chi_square_2x2)	pvalue(fisher_test)	NCM_460(norm)	SW_620(norm)	NCM_460 (raw)	SW_620(raw)	Expression level
hsa-miR-29c-5p_L-2R+3[1] *	ACCGATTTCTCCTGGTGTTCAGA	up|down	inf|0.47	inf|-1.10	2.53E-03|8.03E-03	1.99E-03|9.36E-03	0|64	8|30	0|118	5|66	middle|middle
hsa-miR-375-3p[2]	TTTGTTCGTTCGGCTCGCGTGA	up|down	4.17|0.11	2.06|-3.19	0.00E+00|4.38E-21	0.00E+00|1.98E-24	3,028|157	12,617|17	778|289	8,136|38	high|high
hsa-miR-449a[3]	TGGCAGTGTATTGTTAGCTGGT	up|down	inf|0.15	inf|-2.75	1.34E-04|6.41E-03	8.86E-05|5.80E-03	0|15	12|2	0|28	8|5	middle|middle
hsa-miR-590-3p	TAATTTTATGTATAAGCTAGT	up|down	14.82|0.36	3.89|-1.46	2.23E-63|1.06E-56	4.25E-73|2.56E-59	19|1,313	288|479	5|2,412	186|1,056	middle|middle
hsa-mir-3133-p3	TTGAGTTTTAAGAGTTCTTTAT	up|down	inf|-inf	inf|-inf	5.09E-05|1.12E-04	1.87E-05|3.38E-05	0|18	14|0	0|33	9|0	middle|middle
hsa-miR-9-3p[4]	ATAAAGCTAGATAACCGAAAGT	up|down	3.25|0.05	1.70|-4.28	0.00E+00|3.39E-13	5.42E-98|1.27E-15	268|76	872|4	69|140	562|9	middle|middle
hsa-miR-128-1-5p	CGGGGCCGTAGCACTGTCTGAGA	up|down	inf|0.38	inf|-1.40	3.53E-04|1.92E-03	1.93E-04|1.49E-03	0|54	11|20	0|99	7|45	middle|middle
hsa-miR-140-5p[5, 6]	CAGTGGTTTTACCCTATGGTAG	up|down	3.93|0.30	1.98|-1.73	0.00E+00|6.20E-44	5.22E-223|1.16E-46	428|746	1,684|225	110|1,370	1,086|496	middle|middle
hsa-miR-556-3p_R-1[7] *	ATATTACCATTAGCTCATCTT	up|down	inf|0.21	inf|-2.26	7.49E-06|1.49E-03	1.82E-06|9.00E-04	0|26	17|5	0|48	11|12	middle|middle
hsa-miR-885-5p[8-10]	TCCATTACACTACCCTGCCTCT	up|down	inf|-inf	inf|-inf	9.39E-04|1.53E-02	9.13E-04|1.85E-02	0|7	9|0	0|13	6|0	low|low
hsa-miR-627-3p_L+1[11] *	CTCTTTTCTTTGAGACTCACT	up|down	inf|0.10	inf|-3.35	2.89E-06|3.04E-06	3.83E-07|1.77E-06	0|37	19|4	0|68	12|8	middle|middle

hsa-mir-641-p3[12, 13]	TGACTGTCCTATGTCTTTCCT	up|down	inf|0.09	inf|-3.53	9.36E-11|1.51E-08	6.96E-13|3.54E-09	0|52	36|5	0|96	23|10	middle|middle
hsa-miR-372-3p[14, 15]	AAAGTGCTGCGACATTTGAGCGT	up|down	2.72|-inf	1.45|-inf	3.65E-07|2.63E-03	2.25E-07|1.46E-03	23|11	64|0	6|20	41|0	middle|middle
hsa-miR-576-5p[16, 17]	ATTCTAATTTCTCCACGTCTTT	up|down	inf|0.18	inf|-2.49	2.57E-08|4.85E-23	1.66E-09|1.79E-25	0|224	26|40	0|412	17|88	middle|middle
hsa-miR-625-3p[18, 19]	GACTATAGAACTTTCCCCCTCA	up|down	16.65|0.36	4.06|-1.46	0.00E+00|6.61E-23	0.00E+00|1.01E-23	109|501	1,814|182	28|921	1,170|401	middle|middle
hsa-miR-29b-3p[20, 21]	TAGCACCATTTGAAATCAGTGTT	up|down	2.55|0.48	1.35|-1.06	4.73E-62|2.23E-25	4.37E-63|6.23E-26	288|1,077	735|516	74|1,979	474|1,139	middle|middle
hsa-miR-4521_R+4 *	GCTAAGGAAGTCCTGTGCTCAGTTTT	up|down	inf|0.03	inf|-5.00	2.53E-03|0.00E+00	1.99E-03|0.00E+00	0|4,653	8|145	0|8,545	5|320	high|high
hsa-miR-98-3p_1ss22CT *	CTATACAACTTACTACTTTCCT	up|down	inf|0.16	inf|-2.65	2.17E-14|3.83E-26	1.30E-17|2.43E-29	0|239	50|38	0|439	32|84	middle|middle
hsa-miR-19a-3p[22]	TGTGCAAATCTATGCAAAACTGA	up|down	3.35|0.35	1.75|-1.53	5.33E-88|8.61E-56	5.23E-91|1.10E-58	233|1,186	783|412	60|2,177	505|908	middle|middle
hsa-miR-200b-5p[23-27]	CATCTTACTGGGCAGCATTGGA	up|down	3.02|-inf	1.59|-inf	3.64E-81|1.18E-47	3.44E-83|4.28E-67	265|253	799|0	68|465	515|0	middle|middle
hsa-miR-30d-3p[28]	CTTTCAGTCAGATGTTTGCTGC	up|down	3.49|0.10	1.80|-3.33	4.99E-14|6.66E-32	1.96E-14|4.41E-37	31|236	109|24	8|434	70|52	middle|middle
hsa-miR-548a-5p_R+3[29, 30] *	AAAAGTAATTGCGGTCTTTGGT	up|down	inf|0.15	inf|-2.70	2.53E-03|1.65E-03	1.99E-03|8.36E-04	0|21	8|3	0|38	5|7	middle|middle
hsa-miR-548d-3p[31-33]	CAAAAACCACAGTTTCTTTTGC	up|down	inf|0.22	inf|-2.19	3.27E-02|1.67E-02	4.46E-02|1.01E-02	0|16	4|3	0|29	3|8	middle|middle
hsa-miR-10399-5p_R+1 *	AATTACAGATTGTCTCAGAGAA	up|down	inf|0.07	inf|-3.77	2.57E-08|1.07E-06	1.66E-09|2.30E-07	0|37	26|3	0|68	17|6	middle|middle

hsa-miR-34a-5p[34-36]	TGGCAGTGTCTTAGCTGGTTGT	up|down	2.28|0.28	1.19|-1.84	0.00E+00|1.94E-43	6.26E-151|2.27E-46	918|666	2,097|186	236|1,224	1,352|410	middle|middle
hsa-miR-135b-3p	ATGTAGGGCTAAAAGCCATGGG	up|down	inf|0.07	inf|-3.92	1.31E-19|1.95E-06	2.29E-24|2.24E-07	0|34	70|2	0|63	45|5	middle|middle
hsa-miR-33a-5p_R-1[37-44] *	GTGCATTGTAGTTGCATTGC	up|down	2.09|0.10	1.06|-3.29	1.41E-11|5.55E-10	1.40E-11|5.22E-11	78|66	163|7	20|122	105|15	middle|middle
hsa-miR-181a-3p	ACCATCGACCGTTGATTGTACC	up|down	2.68|0.01	1.42|-6.58	5.01E-19|2.06E-62	4.54E-19|2.97E-84	74|347	198|4	19|637	128|8	middle|middle
hsa-miR-625-5p[45]	AGGGGGAAAGTTCTATAGTCC	up|down	9.39|0.35	3.23|-1.52	0.00E+00|3.72E-06	7.85E-112|3.19E-06	54|103	512|36	14|189	330|79	middle|middle
hsa-miR-15b-3p_R-1 *	CGAATCATTATTTGCTGCTCT	up|down	3.73|0.37	1.90|-1.44	0.00E+00|4.14E-50	8.90E-97|2.11E-52	202|1,176	755|433	52|2,159	487|954	middle|middle
hsa-miR-2467-5p_R+1 *	TGAGGCTCTGTTAGCCTTGGCTCT	up|down	inf|0.18	inf|-2.46	2.53E-03|6.30E-03	1.99E-03|6.20E-03	0|17	8|3	0|32	5|7	middle|middle
hsa-miR-548aa_L-1R-2[46, 47] *	AAAACCACAATTACTTTTGCAC	up|down	inf|0.38	inf|-1.40	2.68E-05|5.63E-03	8.60E-06|5.60E-03	0|43	15|16	0|79	10|36	middle|middle
hsa-miR-31-3p_R+1[48] *	TGCTATGCCAACATATTGCCATC	up|down	2.85|0.15	1.51|-2.70	3.71E-16|1.05E-81	1.39E-16|4.36E-93	54|766	155|117	14|1,406	100|259	middle|middle
hsa-miR-374a-3p	CTTATCAGATTGTATTGTAATT	up|down	6.69|0.32	2.74|-1.66	6.81E-24|3.41E-67	9.38E-26|3.08E-71	19|1,237	130|391	5|2,272	84|862	middle|middle
hsa-miR-23b-5p_L-1[49, 50] *	GGGTTCCTGGCATGCTGATTT	up|down	inf|0.34	inf|-1.55	1.37E-35|1.21E-03	2.65E-45|1.22E-03	0|49	132|17	0|90	85|37	middle|middle
hsa-miR-548ae-5p_R+2 *	AAAAGTAATTGTGGTTTTTGCC	up|down	2.68|0.32	1.42|-1.64	2.66E-04|3.81E-05	2.53E-04|4.18E-05	12|71	33|23	3|131	22|51	middle|middle
hsa-miR-26b-3p_R+1[51, 52] *	CCTGTTCTCCATTACTTGGCTC	up|down	inf|0.47	inf|-1.08	1.69E-07|1.61E-02	1.71E-08|1.43E-02	0|56	23|26	0|102	15|58	middle|middle

<table>
<tr><td>hsa-miR-9-5p[53-56]</td><td>TCTTTGGTTATCTAGCTGTATGA</td><td>up|down</td><td>4.06|0.01</td><td>2.02|-6.72</td><td>0.00E+00|1.39E-32</td><td>6.64E-119|2.68E-43</td><td>214|176</td><td>869|2</td><td>55|323</td><td>561|4</td><td>middle|middle</td></tr>
<tr><td>hsa-miR-454-3p_R+1[57-59] *</td><td>TAGTGCAATATTGCTTATAGGGTT</td><td>up|down</td><td>inf|0.39</td><td>inf|-1.36</td><td>1.12E-87|8.26E-47</td><td>7.34E-114|8.77E-49</td><td>0|1,220</td><td>335|474</td><td>0|2,240</td><td>216|1,046</td><td>middle|middle</td></tr>
<tr><td>hsa-miR-499a-5p[60, 61]</td><td>TTAAGACTTGCAGTGATGTTT</td><td>up|down</td><td>111.34|0.24</td><td>6.80|-2.07</td><td>0.00E+00|9.89E-06</td><td>0.00E+00|1.14E-05</td><td>175|57</td><td>19,499|14</td><td>45|105</td><td>12,574|30</td><td>high|high</td></tr>
<tr><td>hsa-miR-146a-5p[62-66]</td><td>TGAGAACTGAATTCCATGGGTT</td><td>up|down</td><td>12.56|0.04</td><td>3.65|-4.51</td><td>0.00E+00|0.00E+00</td><td>0.00E+00|3.59E-193</td><td>1,442|962</td><td>18,110|42</td><td>371|1,766</td><td>11,679|93</td><td>high|high</td></tr>
<tr><td>hsa-mir-33a-p3</td><td>TGTTCTGGTGGTACCCATG</td><td>up|down</td><td>inf|0.25</td><td>inf|-1.99</td><td>2.53E-03|1.53E-06</td><td>1.99E-03|6.63E-07</td><td>0|72</td><td>8|18</td><td>0|132</td><td>5|40</td><td>middle|middle</td></tr>
<tr><td>hsa-miR-579-3p[67]</td><td>TTCATTTGGTATAAACCGCGATT</td><td>up|down</td><td>inf|0.15</td><td>inf|-2.70</td><td>9.36E-11|1.05E-21</td><td>6.96E-13|1.58E-24</td><td>0|192</td><td>36|29</td><td>0|352</td><td>23|65</td><td>middle|middle</td></tr>
<tr><td>hsa-miR-548n</td><td>CAAAAGTAATTGTGGATTTTGT</td><td>up|down</td><td>inf|0.25</td><td>inf|-2.00</td><td>1.12E-06|5.15E-05</td><td>1.76E-07|4.18E-05</td><td>0|51</td><td>20|13</td><td>0|93</td><td>13|28</td><td>middle|middle</td></tr>
<tr><td>hsa-miR-10399-3p_L+1_1ss22CT *</td><td>TCTCTCGGACAAGCTGTAGGTT</td><td>up|down</td><td>inf|0.08</td><td>inf|-3.68</td><td>3.53E-04|2.67E-06</td><td>1.93E-04|6.93E-07</td><td>0|35</td><td>11|3</td><td>0|64</td><td>7|6</td><td>middle|middle</td></tr>
<tr><td>hsa-miR-29a-3p_R-1[68-72] *</td><td>TAGCACCATCTGAAATCGGTT</td><td>up|down</td><td>2.09|0.44</td><td>1.06|-1.17</td><td>0.00E+00|0.00E+00</td><td>0.00E+00|0.00E+00</td><td>54,704|70,707</td><td>114,101|31,379</td><td>14,057|129,864</td><td>73,580|69,214</td><td>high|high</td></tr>
<tr><td>hsa-miR-374b-3p</td><td>CTTAGCAGGTTGTATTATCATT</td><td>up|down</td><td>inf|0.43</td><td>inf|-1.21</td><td>5.26E-30|2.37E-06</td><td>7.12E-38|2.34E-06</td><td>0|166</td><td>110|72</td><td>0|305</td><td>71|158</td><td>middle|middle</td></tr>
<tr><td>hsa-miR-10401-3p_L+1R-1 *</td><td>GACCTCGCCGTCCCGCCCGCC</td><td>up|down</td><td>10.46|0.48</td><td>3.39|-1.05</td><td>3.84E-66|1.11E-02</td><td>2.41E-74|1.12E-02</td><td>31|66</td><td>326|32</td><td>8|122</td><td>210|71</td><td>middle|middle</td></tr>
<tr><td>hsa-miR-135b-5p[73, 74]</td><td>TATGGCTTTTCATTCCTATGTGA</td><td>up|down</td><td>12.08|0.02</td><td>3.59|-5.37</td><td>0.00E+00|0.00E+00</td><td>0.00E+00|2.39E-217</td><td>245|977</td><td>2,962|24</td><td>63|1,795</td><td>1,910|52</td><td>middle|middle</td></tr>
<tr><td>hsa-miR-577_L+1[75-80] *</td><td>GTAGATAAAATATTGGTACCTG</td><td>up|down</td><td>inf|-inf</td><td>inf|-inf</td><td>0.00E+00|2.68E-27</td><td>2.86E-145|1.07E-37</td><td>0|141</td><td>428|0</td><td>0|259</td><td>276|0</td><td>middle|middle</td></tr>
</table>

hsa-miR-192-3p	CTGCCAATTCCATAGGTCACAG	up|down	inf|-inf	inf|-inf	6.05E-46|2.06E-03	8.21E-59|1.46E-03	0|11	172|0	0|21	111|0	middle|middle

*: passenger strand

Supplementary References

[1] H J, G Z, JH W, et al. Diverse roles of miR-29 in cancer (review) [J]. Oncology reports, 2014, 31(4): 1509-16.

[2] ML S, JS H, DF P, et al. MicroRNA profiles in colorectal carcinomas, adenomas and normal colonic mucosa: variations in miRNA expression and disease progression [J]. Carcinogenesis, 2016, 37(3): 245-61.

[3] X S, S L, P C, et al. miR-449a inhibits colorectal cancer progression by targeting SATB2 [J]. Oncotarget, 2017, 8(60): 100975-88.

[4] L C, JK R, J B, et al. MiR-9, -31, and -182 deregulation promote proliferation and tumor cell survival in colon cancer [J]. Neoplasia (New York, NY), 2012, 14(9): 868-79.

[5] W Z, C Z, L P, et al. MicroRNA-140-5p inhibits the progression of colorectal cancer by targeting VEGFA [J]. Cellular physiology and biochemistry : international journal of experimental cellular physiology, biochemistry, and pharmacology, 2015, 37(3): 1123-33.

[6] L Y, Y L, X H, et al. microRNA -140-5p inhibits colorectal cancer invasion and metastasis by targeting ADAMTS5 and IGFBP5 [J]. Stem cell research & therapy, 2016, 7(1): 180.

[7] FENG C, SUN P, HU J, et al. miRNA-556-3p promotes human bladder cancer proliferation, migration and invasion by negatively regulating DAB2IP expression [J]. International Journal of Oncology, 2017, 50(6): 2101-12.

[8] Z B, W T, G H. miR-885-5p suppresses osteosarcoma proliferation, migration and invasion through regulation of β-catenin.%A Liu Y [J]. Oncology letters, 2019, 17(2): 1996-2004.

[9] Z W, XW W, SL W, et al. MicroRNA-885-5p promotes osteosarcoma proliferation and migration by downregulation of cell division cycle protein 73 homolog expression.%A Yu FQ [J]. Oncology letters, 2019, 17(2): 1565-72.

[10] B Q, F L, Y C, et al. miR-885-5p upregulation promotes colorectal cancer cell proliferation and migration by targeting suppressor of cytokine signaling.%A Su M [J]. Oncology letters, 2018, 16(1): 65-72.

[11] Q Z, YM R, C M, et al. MicroRNA-627 mediates the epigenetic mechanisms of vitamin D to suppress proliferation of human colorectal cancer cells and growth of xenograft tumors in mice.%A Padi SK [J]. Gastroenterology, 2013, 145(2): 437-46.

[12] H Z, L W, P C. miRNA-641 inhibits the proliferation, migration, and invasion and induces apoptosis of cervical cancer cells by directly targeting .%A Yao R [J]. OncoTargets and therapy, 2018, 11(undefined): 8965-76.

[13] N S, J L, N X. miR-641 Functions as a Tumor Suppressor by Targeting MDM2 in Human Lung Cancer.%A Kong Q [J]. Oncology research, 2018, 26(5): 735-41.

[14] PF X, TT G. MiR-372-3p inhibits the growth and metastasis of osteosarcoma cells by targeting FXYD6.%A Xu SY [J]. European review for medical and pharmacological sciences, 2018, 22(1): 62-9.

[15] S L, X Z, Y W, et al. MiR-372-3p promotes cell growth and metastasis by targeting FGF9 in lung squamous cell carcinoma.%A Wang Q [J]. Cancer medicine, 2017, 6(6):

1323-30.
[16] LH Z, G L, M H, et al. MicroRNA-548-3p and MicroRNA-576-5p enhance the migration and invasion of esophageal squamous cell carcinoma cells via NRIP1 down-regulation.%A Ni XF [J]. Neoplasma, 2018, 65(6): 881-7.
[17] ZH C, WC C. Novel circulating microRNAs expression profile in colon cancer: a pilot study.%A Wang YN [J]. European journal of medical research, 2017, 22(1): 51.
[18] R M, Q W, P Z, et al. MiR-625-3p promotes cell migration and invasion via inhibition of SCAI in colorectal carcinoma cells.%A Zheng H [J]. Oncotarget, 2015, 6(29): 27805-15.
[19] D K, W X. MicroRNA-625-3p promotes the proliferation, migration and invasion of thyroid cancer cells by up-regulating astrocyte elevated gene 1.%A Fang L [J]. Biomedicine & pharmacotherapy = Biomedecine & pharmacotherapie, 2018, 102(undefined): 203-11.
[20] C L, T Z, D L, et al. LncRNA H19/miR-29b-3p/PGRN Axis Promoted Epithelial-Mesenchymal Transition of Colorectal Cancer Cells by Acting on Wnt Signaling.%A Ding D [J]. Molecules and cells, 2018, 41(5): 423-35.
[21] O Y, O O, OI C, et al. Differential expressions of cancer-associated genes and their regulatory miRNAs in colorectal carcinoma.%A Kara M [J]. Gene, 2015, 567(1): 81-6.
[22] Z Z, C C, Y L, et al. MicroRNA-19a-3p inhibits breast cancer progression and metastasis by inducing macrophage polarization through downregulated expression of Fra-1 proto-oncogene.%A Yang J [J]. Oncogene, 2014, 33(23): 3014-23.
[23] BR L, JH K, ES C, et al. Effect of young exosomes injected in aged mice [J]. International journal of nanomedicine, 2018, 13(undefined): 5335-45.
[24] K H, M R, J G, et al. Expression profiles of miR-29c, miR-200b and miR-375 in tumour and tumour-adjacent tissues of head and neck cancers [J]. Tumour biology : the journal of the International Society for Oncodevelopmental Biology and Medicine, 2016, 37(9): 12627-33.
[25] K X, C W, N Q, et al. Genetic variants in regulatory regions of microRNAs are associated with lung cancer risk [J]. Oncotarget, 2016, 7(30): 47966-74.
[26] LV R, EC M, HC S, et al. Dual regulation by microRNA-200b-3p and microRNA-200b-5p in the inhibition of epithelial-to-mesenchymal transition in triple-negative breast cancer [J]. Oncotarget, 2015, 6(18): 16638-52.
[27] R C, F B, M D N, et al. microRNAs derived from circulating exosomes as noninvasive biomarkers for screening and diagnosing lung cancer [J]. Journal of thoracic oncology : official publication of the International Association for the Study of Lung Cancer, 2013, 8(9): 1156-62.
[28] K W, M C, W W. Analysis of microRNA (miRNA) expression profiles reveals 11 key biomarkers associated with non-small cell lung cancer [J]. World journal of surgical oncology, 2017, 15(1): 175.
[29] G Z, T W, QK H, et al. MicroRNA-548a-5p promotes proliferation and inhibits apoptosis in hepatocellular carcinoma cells by targeting Tg737 [J]. World journal of gastroenterology, 2016, 22(23): 5364-73.
[30] H P, J T, F R, et al. Chromosomal, epigenetic and microRNA-mediated inactivation of LRP1B, a modulator of the extracellular environment of thyroid cancer cells [J].

Oncogene, 2011, 30(11): 1302-17.
[31] J T, L X, G X. LncRNA MEG3 suppresses migration and promotes apoptosis by sponging miR-548d-3p to modulate JAK-STAT pathway in oral squamous cell carcinoma [J]. IUBMB life, 2019, undefined(undefined): undefined.
[32] Q S, J S, Q W, et al. miR-548d-3p/TP53BP2 axis regulates the proliferation and apoptosis of breast cancer cells [J]. Cancer medicine, 2016, 5(2): 315-24.
[33] H C, JG S, XW C, et al. Preliminary validation of ERBB2 expression regulated by miR-548d-3p and miR-559 [J]. Biochemical and biophysical research communications, 2009, 385(4): 596-600.
[34] Z W, J L, X Z, et al. A three plasma microRNA signature for papillary thyroid carcinoma diagnosis in Chinese patients [J]. Gene, 2019, 693(undefined): 37-45.
[35] YY L, YW T, S G, et al. Cancer-associated fibroblasts contribute to oral cancer cells proliferation and metastasis via exosome-mediated paracrine miR-34a-5p [J]. EBioMedicine, 2018, 36(undefined): 209-20.
[36] M R, C B-S, NP H, et al. Identification of non-invasive miRNAs biomarkers for prostate cancer by deep sequencing analysis of urinary exosomes [J]. Molecular cancer, 2017, 16(1): 156.
[37] J P, C Z, X Z, et al. A two-miRNA signature (miR-33a-5p and miR-128-3p) in whole blood as potential biomarker for early diagnosis of lung cancer [J]. Scientific reports, 2018, 8(1): 16699.
[38] P L, B C, Y G, et al. PNMA1, regulated by miR-33a-5p, promotes proliferation and EMT in hepatocellular carcinoma by activating the Wnt/β-catenin pathway [J]. Biomedicine & pharmacotherapy = Biomedecine & pharmacotherapie, 2018, 108(undefined): 492-9.
[39] JX Y, Y S, L G, et al. Long non-coding RNA DANCR facilitates glioma malignancy by sponging miR-33a-5p [J]. Neoplasma, 2018, 65(5): 790-8.
[40] Z Z, L M, Z G, et al. Long non-coding RNA CASC15 promotes tongue squamous carcinoma progression through targeting miR-33a-5p [J]. Environmental science and pollution research international, 2018, 25(22): 22205-12.
[41] K C, J L, J C, et al. microRNA-33a-5p increases radiosensitivity by inhibiting glycolysis in melanoma [J]. Oncotarget, 2017, 8(48): 83660-72.
[42] N J, X W, X X, et al. lncRNA DANCR promotes tumor progression and cancer stemness features in osteosarcoma by upregulating AXL via miR-33a-5p inhibition [J]. Cancer letters, 2017, 405(undefined): 46-55.
[43] W M, Q S, X C, et al. miR-33a-5p modulates TNF-α-inhibited osteogenic differentiation by targeting SATB2 expression in hBMSCs [J]. FEBS letters, 2016, 590(3): 396-407.
[44] J Z, D W, J X, et al. MicroRNA-33a-5p suppresses growth of osteosarcoma cells and is downregulated in human osteosarcoma [J]. Oncology letters, 2015, 10(4): 2135-41.
[45] H Z, C F, M Z, et al. miR-625-5p/PKM2 negatively regulates melanoma glycolysis state [J]. Journal of cellular biochemistry, 2019, 120(3): 2964-72.

[46] F C, E I, S L, et al. New tumor suppressor microRNAs target glypican-3 in human liver cancer [J]. Oncotarget, 2017, 8(25): 41211-26.
[47] A B, E O-W, K N, et al. Altered Expression of miRNAs Is Related to Larynx Cancer TNM Stage and Patients' Smoking Status [J]. DNA and cell biology, 2017, 36(7): 581-8.
[48] G M, S I, R T, et al. Hsa-miR-31-3p expression is linked to progression-free survival in patients with KRAS wild-type metastatic colorectal cancer treated with anti-EGFR therapy [J]. Clinical cancer research : an official journal of the American Association for Cancer Research, 2014, 20(12): 3338-47.
[49] J B-R, JR L, HB M, et al. Micro-RNA Profiling of Exosomes from Marrow-Derived Mesenchymal Stromal Cells in Patients with Acute Myeloid Leukemia: Implications in Leukemogenesis [J]. Stem cell reviews, 2017, 13(6): 817-25.
[50] U W-E, SH C, AH H, et al. Exosomal onco-miRs from serum of patients with adenocarcinoma of the esophagus: comparison of miRNA profiles of exosomes and matching tumor [J]. Tumour biology : the journal of the International Society for Oncodevelopmental Biology and Medicine, 2015, 36(6): 4643-53.
[51] Y L, L X, Y Z, et al. MiR-26b-3p regulates osteoblast differentiation via targeting estrogen receptor α [J]. Genomics. 2019, 111(5):1089-96.
[52] Q W, C X, Y Z, et al. miR-26b-3p Regulates Human Umbilical Cord-Derived Mesenchymal Stem Cell Proliferation by Targeting Estrogen Receptor [J]. Stem cells and development, 2016, 25(5): 415-26.
[53] J W, B W, H R, et al. miR-9-5p inhibits pancreatic cancer cell proliferation, invasion and glutamine metabolism by targeting GOT1 [J]. Biochemical and biophysical research communications, 2019, 509(1): 241-8.
[54] G L, F W, H Y, et al. MiR-9-5p promotes cell growth and metastasis in non-small cell lung cancer through the repression of TGFBR2 [J]. Biomedicine & pharmacotherapy = Biomedecine & pharmacotherapie, 2017, 96(undefined): 1170-8.
[55] J R, JL V-H, A P, et al. MicroRNA Profile in Patients with Alzheimer's Disease: Analysis of miR-9-5p and miR-598 in Raw and Exosome Enriched Cerebrospinal Fluid Samples [J]. Journal of Alzheimer's disease : JAD, 2017, 57(2): 483-91.
[56] F G, X H, Q S. MicroRNA-9-5p functions as a tumor suppressor in papillary thyroid cancer via targeting BRAF [J]. Oncology letters, 2018, 16(5): 6815-21.
[57] D J, H L, H X, et al. Long Chain Non-Coding RNA (lncRNA) HOTAIR Knockdown Increases miR-454-3p to Suppress Gastric Cancer Growth by Targeting STAT3/Cyclin D1 [J]. Medical science monitor : international medical journal of experimental and clinical research, 2019, 25(undefined): 1537-48.
[58] C J, T L, L S. Downregulation of Calbindin 1 by miR-454-3p Suppresses Cell Proliferation in Nonsmall Cell Lung Cancer In Vitro [J]. Cancer biotherapy & radiopharmaceuticals, 2019, 34(2): 119-27.
[59] N S, L X, R W, et al. miR-454-3p Is an Exosomal Biomarker and Functions as a Tumor Suppressor in Glioma [J]. Molecular cancer therapeutics, 2019, 18(2): 459-69.
[60] JC S, B Z, RX S, et al. MiR-499a-5p suppresses apoptosis of human nucleus pulposus cells and degradation of their extracellular matrix by targeting SOX4 [J]. Biomedicine & pharmacotherapy = Biomedecine & pharmacotherapie, 2019, 113(undefined): 108652.
[61] J L, L H, P S, et al. MicroRNA-499a-5p inhibits osteosarcoma cell proliferation and differentiation by targeting protein phosphatase 1D through protein kinase

B/glycogen synthase kinase 3β signaling [J]. Oncology letters, 2018, 15(4): 4113-20.
[62] DL Y, BB S, J L, et al. MiR-146a-5p level in serum exosomes predicts therapeutic effect of cisplatin in non-small cell lung cancer [J]. European review for medical and pharmacological sciences, 2017, 21(11): 2650-8.
[63] Y T, W A, A S, et al. Small RNAs detected in exosomes derived from the MH7A synovial fibroblast cell line with TNF-α stimulation [J]. PloS one, 2018, 13(8): e0201851.
[64] JP L, LF D, FF C, et al. miR-146a-5p targets interleukin-1 receptor-associated kinase 1 to inhibit the growth, migration, and invasion of breast cancer cells [J]. Oncology letters, 2019, 17(2): 1573-80.
[65] W G, J H, Z J, et al. Expression of miR-146a-5p in breast cancer and its role in proliferation of breast cancer cells [J]. Oncology letters, 2018, 15(6): 9884-8.
[66] YL L, J W, CY Z, et al. MiR-146a-5p inhibits cell proliferation and cell cycle progression in NSCLC cell lines by targeting CCND1 and CCND2 [J]. Oncotarget, 2016, 7(37): 59287-98.
[67] L F, R M, M A, et al. miR-579-3p controls melanoma progression and resistance to target therapy [J]. Proceedings of the National Academy of Sciences of the United States of America, 2016, 113(34): E5005-13.
[68] J C, N G, B Q, et al. OSCC-derived exosomes promote M2-type macrophage polarization mediated by exosome-enclosed miR-29a-3p [J]. American journal of physiology Cell physiology, 2019, undefined(undefined): undefined.
[69] Y M, Y S. miR-29a-3p inhibits growth, proliferation, and invasion of papillary thyroid carcinoma by suppressing NF-κB signaling via direct targeting of OTUB2 [J]. Cancer management and research, 2019, 11(undefined): 13-23.
[70] X W, S L, L C, et al. miR-29a-3p suppresses cell proliferation and migration by downregulating IGF1R in hepatocellular carcinoma [J]. Oncotarget, 2017, 8(49): 86592-603.
[71] J S, E L, L L, et al. MiR-29a-3p suppresses cell proliferation in laryngocarcinoma by targeting prominin 1 [J]. FEBS open bio, 2017, 7(5): 645-51.
[72] Z Z, L W, W S, et al. Reduced miR-29a-3p expression is linked to the cell proliferation and cell migration in gastric cancer [J]. World journal of surgical oncology, 2015, 13(undefined): 101.
[73] Z Z, X C, N Y, et al. miR-135b-5p Promotes migration, invasion and EMT of pancreatic cancer cells by targeting NR3C2 [J]. Biomedicine & pharmacotherapy = Biomedecine & pharmacotherapie, 2017, 96(undefined): 1341-8.
[74] M L, Y H, W S, et al. miR-135b-5p promotes gastric cancer progression by targeting CMTM3 [J]. International journal of oncology, 2018, 52(2): 589-98.
[75] C Y, Q M, X P, et al. MiR-577 suppresses epithelial-mesenchymal transition and metastasis of breast cancer by targeting Rab25 [J]. Thoracic cancer, 2018, 9(4): 472-9.
[76] B W, L S, J L, et al. miR-577 suppresses cell proliferation and epithelial-mesenchymal transition by regulating the WNT2B mediated Wnt/β-catenin pathway in non-small cell lung cancer [J]. Molecular medicine reports, 2018, 18(3): 2753-61.

[77] KC X, DD H, L Z, et al. MiR-577 inhibits papillary thyroid carcinoma cell proliferation, migration and invasion by targeting SphK2 [J]. European review for medical and pharmacological sciences, 2017, 21(17): 3794-800.
[78] W Z, C S, C L, et al. miR-577 inhibits glioblastoma tumor growth via the Wnt signaling pathway [J]. Molecular carcinogenesis, 2016, 55(5): 575-85.
[79] LY W, B L, HH J, et al. Inhibition effect of miR-577 on hepatocellular carcinoma cell growth via targeting β-catenin [J]. Asian Pacific journal of tropical medicine, 2015, 8(11): 923-9.
[80] H J, H J, L Z, et al. microRNA-577 suppresses tumor growth and enhances chemosensitivity in colorectal cancer [J]. Journal of biochemical and molecular toxicology, 2017, 31(6): undefined.
[81] D Y, Y M, D W, et al. Prognostic Role of Circulating Exosomal miR-425-3p for the Response of NSCLC to Platinum-Based Chemotherapy [J]. Cancer epidemiology, biomarkers & prevention : a publication of the American Association for Cancer Research, cosponsored by the American Society of Preventive Oncology, 2019, 28(1): 163-73.
[82] T F, H L, G L, et al. Tumor-derived exosomal miR-1247-3p induces cancer-associated fibroblast activation to foster lung metastasis of liver cancer [J]. Nature communications, 2018, 9(1): 191.
[83] N M, MT V, R G-C, et al. Tumor microRNA expression profiling identifies circulating microRNAs for early breast cancer detection [J]. Clinical chemistry, 2015, 61(8): 1098-106.
[84] M L, Z Q, X M, et al. MiR-628-5p decreases the tumorigenicity of epithelial ovarian cancer cells by targeting at FGFR2 [J]. Biochemical and biophysical research communications, 2018, 495(2): 2085-91.
[85] A S, H G, A D, et al. Circulatory miR-628-5p is downregulated in prostate cancer patients [J]. Tumour biology : the journal of the International Society for Oncodevelopmental Biology and Medicine, 2014, 35(5): 4867-73.
[86] X Q, S Y, X X, et al. Comparative analysis of microRNA expression profiles between A549, A549/DDP and their respective exosomes [J]. Oncotarget, 2017, 8(26): 42125-35.
[87] B J, L X, F C. The role of miR-766-5p in cell migration and invasion in colorectal cancer [J]. Experimental and therapeutic medicine, 2018, 15(3): 2569-74.
[88] K W, R Y, T O. MicroRNA-664a-5p promotes neuronal differentiation of SH-SY5Y cells [J]. Genes to cells : devoted to molecular & cellular mechanisms, 2018, 23(3): 225-33.
[89] TH Y, C Q, J S, et al. MiR-877-5p suppresses cell growth, migration and invasion by targeting cyclin dependent kinase 14 and predicts prognosis in hepatocellular carcinoma [J]. European review for medical and pharmacological sciences, 2018, 22(10): 3038-46.
[90] X J, Y C, H C, et al. Evaluation of Tumor-Derived Exosomal miRNA as Potential Diagnostic Biomarkers for Early-Stage Non-Small Cell Lung Cancer Using Next-Generation

Sequencing [J]. Clinical cancer research : an official journal of the American Association for Cancer Research, 2017, 23(17): 5311-9.
[91] L T, Z Z, L X, et al. MiR-361-5p inhibits the mobility of gastric cancer cells through suppressing epithelial-mesenchymal transition via the Wnt/β-catenin pathway [J]. Gene, 2018, 675(undefined): 102-9.
[92] J L, J Y, L Y, et al. miR-361-5p inhibits glioma migration and invasion by targeting SND1 [J]. OncoTargets and therapy, 2018, 11(undefined): 5239-52.
[93] X Z, R S, W G, et al. Inhibition of miR-361-5p suppressed pulmonary artery smooth muscle cell survival and migration by targeting ABCA1 and inhibiting the JAK2/STAT3 pathway [J]. Experimental cell research, 2018, 363(2): 255-61.
[94] J H, J Y, Y D, et al. Overexpression of miR-361-5p in triple-negative breast cancer (TNBC) inhibits migration and invasion by targeting RQCD1 and inhibiting the EGFR/PI3K/Akt pathway [J]. Bosnian journal of basic medical sciences, 2019, 19(1): 52-9.
[95] F M, L Z, L M, et al. MiR-361-5p inhibits glycolytic metabolism, proliferation and invasion of breast cancer by targeting FGFR1 and MMP-1 [J]. Journal of experimental & clinical cancer research : CR, 2017, 36(1): 158.
[96] L T, Z Z, L X, et al. MiR-361-5p suppresses chemoresistance of gastric cancer cells by targeting FOXM1 via the PI3K/Akt/mTOR pathway [J]. Oncotarget, 2018, 9(4): 4886-96.
[97] XW H, X S, Y Y, et al. miR-361-5p suppresses lung cancer cell lines progression by targeting FOXM1 [J]. Neoplasma, 2017, 64(4): 526-34.
[98] W C, Y L, K X, et al. miR-361-5p inhibits hepatocellular carcinoma cell proliferation and invasion by targeting VEGFA [J]. Biochemical and biophysical research communications, 2016, 479(4): 901-6.
[99] ZG C, YN H, L Y, et al. Positive expression of miR-361-5p indicates better prognosis for breast cancer patients [J]. Journal of thoracic disease, 2016, 8(7): 1772-9.
[100] ZL Z, FM T, CL S. Downregulation of miR-361-5p associates with aggressive clinicopathological features and unfavorable prognosis in non-small cell lung cancer [J]. European review for medical and pharmacological sciences, 2016, 20(24): 5132-6.
[101] F M, H S, B G, et al. MiR-361-5p inhibits colorectal and gastric cancer growth and metastasis by targeting staphylococcal nuclease domain containing-1 [J]. Oncotarget, 2015, 6(19): 17404-16.
[102] D L, T T, B X, et al. MiR-361-5p acts as a tumor suppressor in prostate cancer by targeting signal transducer and activator of transcription-6(STAT6) [J]. Biochemical and biophysical research communications, 2014, 445(1): 151-6.
[103] Y F, X L, Q C, et al. Downregulated miR-98-5p promotes PDAC proliferation and metastasis by reversely regulating MAP4K4 [J]. Journal of experimental & clinical cancer research : CR, 2018, 37(1): 130.
[104] F W, Q M, X W, et al. NEAT1/hsa-mir-98-5p/MAPK6 axis is involved in non-small-cell lung cancer development [J]. Journal of cellular biochemistry, 2019, 120(3): 2836-46.

[105] T J, M L, Q L, et al. MicroRNA-98-5p Inhibits Cell Proliferation and Induces Cell Apoptosis in Hepatocellular Carcinoma via Targeting IGF2BP1 [J]. Oncology research, 2017, 25(7): 1117-27.

[106] YF L, JR Y, Z Y, et al. Promoter hypomethylation mediated upregulation of MicroRNA-10b-3p targets FOXO3 to promote the progression of esophageal squamous cell carcinoma (ESCC) [J]. Journal of experimental & clinical cancer research : CR, 2018, 37(1): 301.

[107] L G, D J, N L, et al. Up-regulation of miR-10b-3p promotes the progression of hepatocellular carcinoma cells via targeting CMTM5 [J]. Journal of cellular and molecular medicine, 2018, 22(7): 3434-41.

[108] M Z, Z H, D Z, et al. A panel of microRNA signature in serum for colorectal cancer diagnosis [J]. Oncotarget, 2017, 8(10): 17081-91.

[109] Z Z, M W, J G, et al. Clinical value of miR-425-5p detection and its association with cell proliferation and apoptosis of gastric cancer [J]. Pathology, research and practice, 2017, 213(8): 929-37.

[110] J Q, Y L, X P, et al. Oncogenic miR-425-5p is associated with cellular migration, proliferation and apoptosis in renal cell carcinoma [J]. Oncology letters, 2018, 16(2): 2175-84.

[111] F F, T S, T Z, et al. MiR-425-5p promotes invasion and metastasis of hepatocellular carcinoma cells through SCAI-mediated dysregulation of multiple signaling pathways [J]. Oncotarget, 2017, 8(19): 31745-57.

[112] YF Y, FM G, BS W, et al. MiR-425-5p promotes tumor progression via modulation of CYLD in gastric cancer [J]. European review for medical and pharmacological sciences, 2017, 21(9): 2130-6.

[113] L S, R J, J L, et al. MicoRNA-425-5p is a potential prognostic biomarker for cervical cancer [J]. Annals of clinical biochemistry, 2017, 54(1): 127-33.

[114] JY Z, XP S, YN L, et al. MicroRNA-425-5p promotes the development of prostate cancer via targeting forkhead box J3 [J]. European review for medical and pharmacological sciences, 2019, 23(2): 547-54.

[115] Z Z, Y L, L F, et al. microRNA-425-5p is upregulated in human gastric cancer and contributes to invasion and metastasis and [J]. Experimental and therapeutic medicine, 2015, 9(5): 1617-22.

[116] M C, AA D, S S, et al. MicroRNA-466 inhibits tumor growth and bone metastasis in prostate cancer by direct regulation of osteogenic transcription factor RUNX2 [J]. Cell death & disease, 2017, 8(1): e2572.

[117] W C, L F, S T, et al. MicroRNA-466 inhibits osteosarcoma cell proliferation and induces apoptosis by targeting CCND1 [J]. Experimental and therapeutic medicine, 2018, 16(6): 5117-22.

[118] F T, Y Y, H P, et al. MicroRNA-466 (miR-466) functions as a tumor suppressor and prognostic factor in colorectal cancer (CRC) [J]. Bosnian journal of basic medical sciences, 2018, 18(3): 252-9.

[119] P S, Y S, JM G, et al. A New MicroRNA Expression Signature for Cervical Cancer [J]. International journal of gynecological cancer : official journal of the International Gynecological Cancer Society, 2017, 27(2): 339-43.

[120] L W, B L, L Z, et al. miR-664a-3p functions as an oncogene by targeting Hippo pathway in the development of gastric cancer [J]. Cell proliferation, 2019, undefined(undefined): e12567.

[121] A K, N P, M A, et al. Antiproliferative and Pro-Apoptotic Effects of MiR-4286 Inhibition in Melanoma Cells [J]. PloS one, 2016, 11(12): e0168229.

[122] H X, C W, Z L. miR-196a-5p promotes metastasis of colorectal cancer via targeting IκBα [J]. BMC cancer, 2019, 19(1): 30.

[123] MH C, Y Y, J K, et al. miR-550a-3-5p acts as a tumor suppressor and reverses BRAF inhibitor resistance through the direct targeting of YAP [J]. Cell death & disease, 2018, 9(6): 640.

[124] Y L, XL Z, XF L, et al. miR-212-3p reduced proliferation, and promoted apoptosis of fibroblast-like synoviocytes via down-regulating SOX5 in rheumatoid arthritis [J]. European review for medical and pharmacological sciences, 2018, 22(2): 461-71.

[125] H L, C L, C S, et al. MiR-212-3p inhibits glioblastoma cell proliferation by targeting SGK3 [J]. Journal of neuro-oncology, 2015, 122(3): 431-9.

[126] Y S, X Y, R D, et al. miR-130b-3p inhibits cell invasion and migration by targeting the Notch ligand Delta-like 1 in breast carcinoma [J]. Gene, 2017, 609(undefined): 80-7.

[127] M D, KV G, Y T, et al. Plasma exosomal miRNAs-based prognosis in metastatic kidney cancer [J]. Oncotarget, 2017, 8(38): 63703-14.

[128] Y G, R C, C Z, et al. miR-132-3p boosts caveolae-mediated transcellular transport in glioma endothelial cells by targeting PTEN/PI3K/PKB/Src/Cav-1 signaling pathway [J]. FASEB journal : official publication of the Federation of American Societies for Experimental Biology, 2019, 33(1): 441-54.

[129] P Z, C F, J D, et al. Association of miR-1247-5p expression with clinicopathological parameters and prognosis in breast cancer [J]. International journal of experimental pathology, 2018, 99(4): 199-205.

[130] B Z, Y L, Y F, et al. Downregulated miR-1247-5p associates with poor prognosis and facilitates tumor cell growth via DVL1/Wnt/β-catenin signaling in breast cancer [J]. Biochemical and biophysical research communications, 2018, 505(1): 302-8.

[131] Y C, W F, W G, et al. miR-1247-5p functions as a tumor suppressor in human hepatocellular carcinoma by targeting Wnt3 [J]. Oncology reports, 2017, 38(1): 343-51.

[132] J P, F J, J Z, et al. HSP90: A Novel Target Gene of miRNA-628-3p in A549 Cells [J]. BioMed research international, 2018, 2018(undefined): 4149707.

[133] L C, X J, H C, et al. microRNA-628 inhibits the proliferation of acute myeloid leukemia cells by directly targeting IGF-1R [J]. OncoTargets and therapy, 2019, 12(undefined): 907-19.
